# Spatial and multi‐omic profiling reveals pericyte‐derived CCL19 as a key prognostic factor in CNS lymphoma

**DOI:** 10.1002/hem3.70412

**Published:** 2026-06-22

**Authors:** Julia C. Kuehn, Lauritz Miarka, Roman Sankowski, Jurik Mutter, Nicolas N. Neidert, Elena Grabis, Junyi Zhang, Christian Klingler, Fabian Hummel, Lavanya Ranganathan, Sabine Bleul, Eliza M. Lauer, Dieter H. Heiland, Katharina Müller, Hans C. Reinhardt, Sascha Dietrich, Gerald Illerhaus, Louisa von Baumgarten, Stefan Alig, Maximilian Diehn, Bastian E. A. Sajonz, Jürgen Beck, Volker A. Coenen, Marco Prinz, Elisabeth Schorb, Ash A. Alizadeh, Justus Duyster, Peter Reinacher, Florian Scherer

**Affiliations:** ^1^ Department of Medicine I, Medical Center – University of Freiburg, Faculty of Medicine University of Freiburg Freiburg Germany; ^2^ Institute of Neuropathology, Faculty of Medicine University of Freiburg Freiburg Germany; ^3^ German Cancer Consortium (DKTK) Partner Site Freiburg and German Cancer Research Center (DKFZ) Heidelberg Germany; ^4^ Department of Medicine, Divisions of Oncology and Hematology Stanford University Stanford California USA; ^5^ Department of Neurosurgery, Medical Center – University of Freiburg, Faculty of Medicine University of Freiburg Freiburg Germany; ^6^ Department of Neurosurgery, University Hospital Erlangen Friedrich‐Alexander University Erlangen‐Nürnberg Erlangen Germany; ^7^ Department of Neurological Surgery, Lou and Jean Malnati Brain Tumor Institute, Robert H. Lurie Comprehensive Cancer Center, Feinberg School of Medicine Northwestern University Chicago Illinois USA; ^8^ Department of Neurology, University Hospital LMU Munich Munich Germany; ^9^ Department of Hematology and Stem Cell Transplantation University Hospital Essen, University Duisburg‐Essen, West German Cancer Center, German Cancer Consortium (DKTK Partner Site Essen), Center for Molecular Biotechnology Essen Germany; ^10^ Department of Hematology, Oncology and Clinical Immunology University Hospital Düsseldorf Düsseldorf Germany; ^11^ Clinic of Hematology, Oncology, Stem Cell Transplantation and Palliative Care, Klinikum Stuttgart Stuttgart Germany; ^12^ Department of Radiation Oncology Stanford University Medical Center Stanford California USA; ^13^ Department of Stereotactic and Functional Neurosurgery, Medical Center – University of Freiburg, Faculty of Medicine University of Freiburg Freiburg Germany; ^14^ Signalling Research Centres BIOSS and CIBSS University of Freiburg Freiburg Germany; ^15^ Fraunhofer Institute for Laser Technology (ILT) Aachen Germany

## Abstract

Biological mechanisms underlying clinical heterogeneity in central nervous system lymphoma (CNSL) are largely unknown. While previous studies suggest the chemokine CLL19 as a crucial factor for the formation of CNSL in murine models, its role in human disease remains elusive. Here, we performed in‐depth genetic and transcriptomic profiling of 82 CNSL specimens and identified distinct genetic aberrations and tumor cell compositions in lymphomas with high CCL19 expression, both of which were associated with immunosuppressive and anti‐apoptotic signatures. CCL19 levels varied widely across CNSL patients. High CCL19 expression was significantly and independently associated with inferior progression‐free and overall survival. Spatial and single‐nucleus analyses as well as immunohistochemistry revealed pericytes within vessel‐rich areas as the predominant source of CCL19, accompanied by significant co‐localization of CCL19 with its primary receptor CCR7 that was enriched in plasmablast‐like malignant B cells, as well as dendritic cells, NK cells, and CD4^+^ T cells. Collectively, our study identified pericyte‐derived CCL19 as a novel prognostic marker in CNSL that is associated with unfavorable genetic aberrations and modifications of the immune landscape towards a resting tumor microenvironment. Spatial CCR7 co‐localization suggests avenues for future therapeutic strategies targeting the CCL19–CCR7 axis.

## INTRODUCTION

Central nervous system lymphomas (CNSLs) are aggressive non‐Hodgkin lymphomas confined to the CNS compartment and classified as large B‐cell lymphomas (LBCLs) of immune‐privileged sites, which can present as primary CNSL (PCNSL) or secondary CNS involvement of systemic LBCL.^1^ Over the past decade, treatment options for this extranodal B‐cell lymphoma type have significantly improved, with high cure rates achieved through methotrexate‐based immunochemotherapies. Despite these advances, a substantial fraction of patients still experience disease progression, and relapsed/refractory CNSL remains a therapeutic challenge associated with poor overall survival (OS).^2^, ^3^, ^4^

The molecular and immunological mechanisms underlying clinical heterogeneity and treatment failure in CNSL remain poorly understood. Previous work in murine models introduced astrocyte‐derived CCL19 as a key factor mediating parenchymal retention of malignant B cells and promoting CNSL formation in the brain.^5^ In systemic B‐cell lymphomas, the interaction between CCL19 and its chemokine receptor CCR7 on lymphoma cells facilitates lymph node homing and extranodal tissue infiltration.^6^ Yet, the biological role of CCL19 within the unique CNSL microenvironment and its clinical significance in human disease remain unexplored. In this study, we conducted in‐depth multi‐omics and single‐cell profiling of 82 PCNSL tissue specimens to elucidate the cellular and spatial origin of CCL19 expression, its impact on the lymphoma microenvironment (LME) and immune cell landscape, its prognostic relevance, and association with clinicopathological features in brain lymphoma patients.

## MATERIALS AND METHODS

### Patient cohort and tissue collection

Tumor specimens were obtained by stereotactic biopsy or resection from 92 patients treated at the University Medical Centers Freiburg and Munich (Germany) for CNSL (*n* = 82; PCNSL *n* = 70; secondary CNSL [SCNSL] *n* = 12) and glioblastoma (GBM, *n* = 10), which we included as a reference tumor arising within the same anatomical compartment. Patients provided written informed consent for the collection of biospecimens and molecular analyses performed in this study in accordance with the Declaration of Helsinki and approved by the local ethics committees (DRKS00015307, 23‐1234‐S1, 22‐0008). Further details for the collection and processing of biospecimens are provided in the Supporting Information S1. The median follow‐up of the whole cohort was 488 days (range: 25–4411 days) and 575 days (range: 25–4411 days) for patients treated with curative intent. All patients underwent routine diagnostic procedures and treatment according to study protocols where applicable (ClinicalTrials.gov identifier: NCT02531841 or DRKS00011932) or institutional standards and national/international guidelines. Further details are provided in the Supporting Information S1. Radiographic studies were performed as part of routine clinical care, and treatment response was assessed according to the International PCNSL Collaborative Group (IPCG) criteria.^7^ Furthermore, magnetic resonance imaging (MRI) 3D tumor volumes were measured at the baseline (for details see Supporting Information S1).^8^ Detailed patient characteristics including subject demographics, the distribution of age and sex, as well as treatment information are provided in Supporting Information S2: Tables 1–3.

### Bulk tumor RNA sequencing

To perform bulk RNA sequencing of CNSL and GBM specimens, RNA was isolated from FFPE or fresh frozen tumor tissue, followed by ribosomal RNA depletion (see Supporting Information S1). After library preparation, RNA sequencing and data analysis were performed as previously described, including raw count data normalization using the median‐of‐ratios method implemented in DESeq2, with detailed information provided in the Supporting Information S1.^9^, ^10^ Using the RNA sequencing datasets, CNSL ecotypes and cell states were defined using the Lymphoma EcoTyper.^11^ To estimate the cellular composition based on the gene expression data, the deconvolution tool CIBERSORTx was applied.^12^ Furthermore, RNA sequencing datasets as well as survival data were obtained from 42 DLBCL patients from The Cancer Genome Atlas Program (TCGA, dbGaP accession: phs000178) and from 24 GBM patients from the Ivy Glioblastoma Atlas Project.^13^

### Targeted capture sequencing and shallow whole‐genome sequencing

Cancer Personalized Profiling by Deep Sequencing (CAPP‐Seq) was performed to profile the mutational landscape of CNSL tumors. DNA from plasma‐depleted whole blood (PDWB) served as germline controls. DNA isolation from tumor and PDWB as well as library preparation and sequencing were performed as described before and detailed in the Supporting Information S1.^14^ Tumor variants were identified using a custom genotyping pipeline as previously reported.^14^ The tumor genotyping profiles were used for LymphGen classification.^15^ Libraries generated for the CAPP‐Seq workflow were further utilized for shallow whole‐genome sequencing (sWGS). Raw sequencing data were processed using ichorCNA and GISTIC2 to generate genome‐wide copy number aberration profiles for bin sizes of 50 and 500 kb.^16^, ^17^ Further details are provided in the Supporting Information S1.

### Single‐nucleus RNA sequencing

Fresh frozen tissue from ten CNSL patients, stored in liquid nitrogen, was used to prepare single nuclei suspensions to perform single‐nucleus RNA sequencing on the 10x Chromium Single Cell platform. The detailed experimental workflow is provided in the Supporting Information S1. In brief, nuclei were loaded onto a Chromium X Controller (10x Genomics, Pleasanton, CA, USA) using the Next GEM Single Cell 3′ Reagent Kit v3.1 and the GEM‐X Universal 3′ Gene Expression Reagent Kit v4. Library construction with sample indexing was performed according to the manufacturer's instructions. Sequencing was carried out on the Illumina NextSeq 1000 platform to a depth of approximately 20,000 reads per cell. After sequencing, reads were aligned to the human reference (GRCh38‐2020‐A), single nuclei were identified, and expression levels of individual genes per nucleus were defined using the CellRanger workflow (v.7.2.0 and v.8.0). The resulting filtered count matrices were further analyzed by the Seurat 5.1 package (Supporting Information S1).^18^ After integration of all single nuclei datasets, dimensionality reduction, clustering, cluster annotation, and characterization of subclusters (i.e., the B‐cell subcluster and vascular cluster) were performed using various pipelines within Seurat 5.1 and Ucell (Supporting Information S1).^19^

### Spatial transcriptomics

We performed spatial transcriptomics for nine CNSL tumor samples, using two different 10x Visium workflows, which are described in detail in the Supporting Information S1. Following these workflows, all generated libraries were sequenced on the Illumina NextSeq 1000 platform, and the resulting sequencing as well as imaging data were processed using the Space Ranger Pipeline v2.1 standard workflow. To further analyze the resulting datasets, we applied the standard workflow of SPATA2.^20^ Segmentation of images into “vessel‐poor” and “vessel‐rich” areas was based on hematoxylin and eosin (H&E) images and conducted by an independent neuropathologist. Using the createSpatialSegmentation() function, all vascularized structures were surrounded, and spots within these areas were defined as “vessel‐rich.” Areas with visible artifacts, such as tissue folds or neuronal regions lacking detectable lymphoma infiltration, were excluded. All remaining spots were classified as “vessel‐poor.” In addition, to determine the CCL19 expression over distance from “vessel‐rich” to “vessel‐poor” areas, a trajectory was drawn using the createSpatialTrajectories() function of the SPATA2 package.^20^ To systematically assess and quantify whether CCL19 was enriched in vascularized tumor regions and co‐localized with CCR7, we applied bootstrapping approaches that are outlined with full methodological details in the Supporting Information S1.

### Fluorescent immunohistochemistry

Fluorescent immunohistochemistry of human PCNSL specimens was performed as previously described.^21^ Briefly, FFPE tissue sections were deparaffinized and subjected to heat‐induced antigen retrieval (citrate buffer pH 6.0). Then, sections were blocked and permeabilized with phosphate‐buffered saline (PBS) containing 5% bovine serum albumin and 0.3% Triton‐X for 90 min at room temperature. Primary antibodies against CCL19 (1:50; AF880, Bio‐Techne, Minneapolis, MN, USA) and PDGFRα/β (1:100; ab215978, Abcam, Cambridge, UK) as well as CD20 (Ready‐to‐use; L26, Agilent Technologies, Santa Clara, USA), CD3 (1:200; CD3‐12, Bio‐Rad, Feldkirchen, DE), CD31 (Ready‐to‐use; JC70A, Agilent Technologies, Santa Clara, USA), and GFAP (Ready‐to‐use; GA52461‐2, Agilent Technologies, Santa Clara, USA) were applied to the same tissue sections in single and co‐staining approaches. All primary antibodies were incubated overnight at 4°C. After three washes with PBS, secondary antibodies were added as follows: Alexa Fluor 488 (1:500) or Alexa Fluor 647 (1:500; both Thermo Fisher Scientific) for 3 h at room temperature. Nuclei were counterstained with DAPI for 20 min. Images were acquired using the conventional fluorescence microscope BZ‐9000 (Keyence) and processed in ImageJ/Fiji (v1.54g). H&E staining was performed on adjacent sections using standard protocols. Double‐positive cells were quantified relative to all CCL19^+^ cells within each region of interest. A total of at least nine independent regions of interest across all four cases were analyzed, and values were averaged across all regions of interest.

### Statistical analysis

Continuous variables were presented as median and 25th–75th percentiles, and compared using the Mann–Whitney *U* test. Categorial variables were compared using the *Χ* ^2^ test. Correlations of variables were determined using the Spearman correlation coefficient. Benjamini–Hochberg correction was performed for multiple hypothesis testing. Time‐to‐event variables were visualized using the Kaplan–Meier method. Log‐rank tests were used to evaluate survival differences. Further survival associations were analyzed using Cox proportional‐hazards regression. Hazard ratios (HRs) were normalized and expressed as *z*‐scores. Progression‐free survival (PFS) and OS were estimated according to the IPCG response criteria. Only patients undergoing curative‐intent immunochemotherapy were considered for survival analyses. Statistical tests were performed using GraphPad Prism (version 9.1.1) and the R survival package. P‐values < 0.05 were considered as significant. Benjamini–Hochberg correction was performed for multiple hypothesis testing as noted.

## RESULTS

### CCL19 expression in CNSL and its prognostic relevance

**Figure 1:**
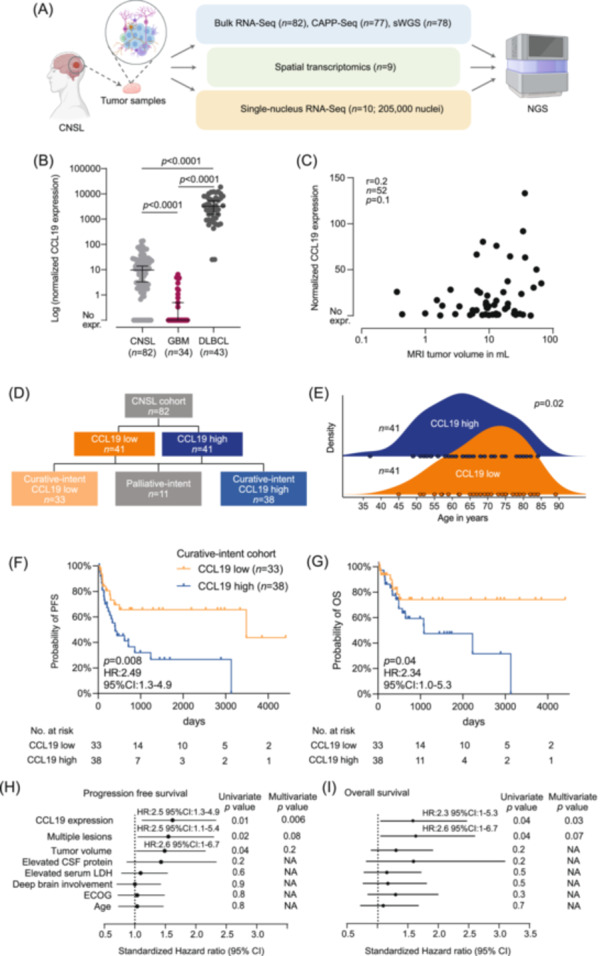
**Clinical relevance of CCL19 in central nervous system lymphoma (CNSL)**. **(A)** Schematic overview of the experimental workflow. Tumor samples from a cohort of 82 CNSL patients were analyzed using a multi‐omics approach with bulk RNA sequencing (RNA‐Seq, *n* = 82), target capture sequencing (CAPP‐Seq, *n* = 77), and shallow whole‐genome sequencing (sWGS, *n* = 78). Additionally, a spatial transcriptomics dataset of 9 tumor samples and a single‐nucleus RNA sequencing dataset consisting of 205,000 nuclei from 10 distinct samples were generated. NGS, next‐generation sequencing. **(B)** Comparison of CCL19 expression levels between CNSL, glioblastoma, and systemic diffuse large B‐cell lymphoma. Bold lines represent the median, and error bars show the 95% confidence intervals. DLBCL, diffuse large B‐cell lymphoma; expr., expression; GBM, glioblastoma. **(C)** Scatter plot showing the correlation of normalized CCL19 expression levels and magnetic resonance imaging (MRI) tumor volumes. mL, milliliters. **(D)** Flowchart demonstrating patient cohorts. **(E)** Age distribution of patients in the “CCL19 high” and “CCL19 low” subgroups. Dots showing the individual values. Kaplan–Meier analyses of **(F)** progression‐free survival (PFS) and **(G)** overall survival (OS) in patients with high CCL19 expression (blue) and low CCL19 expression (orange) treated with curative‐intent therapy. CI, confidence interval; HR, hazard ratio; No., number. Forest plots showing standardized hazard ratios for **(H)** PFS and **(I)** OS estimated by univariate and multivariate Cox proportional‐hazards regression outcome analyses, incorporating CCL19 expression and key clinical and radiographic indices. CSF, cerebrospinal fluid; ECOG, Eastern Cooperative Oncology Group score; LDH, lactate dehydrogenase.

We identified tumor specimens from 82 patients at initial CNSL diagnosis for comprehensive genomic and transcriptomic profiling using RNA sequencing, targeted capture sequencing (CAPP‐Seq), and sWGS (Figure 1A, Supporting Information S2: Table 1, and Supporting Information S1: Methods). CCL19 expression levels varied widely across this cohort, with 13% of samples (11/82) showing no detectable CCL19 expression (Figure 1B). Notably, CCL19 levels were significantly higher in CNSL compared to GBM (P = 1.4e−10), while CCL19 expression was substantially lower compared to systemic diffuse LBCL (DLBCL [TCGA cohort, dbGaP accession: phs000178], P = 2.2e−16, Figure 1B). Further, the abundance of CCL19 tended to correlate with tumor volumes measured by 3D MRI, supporting previous findings from murine models that CCL19 contributes to the retention of lymphoma cells within the brain parenchyma (*r* = 0.2, P = 0.1, Figure 1C).^5^

**Figure 2:**
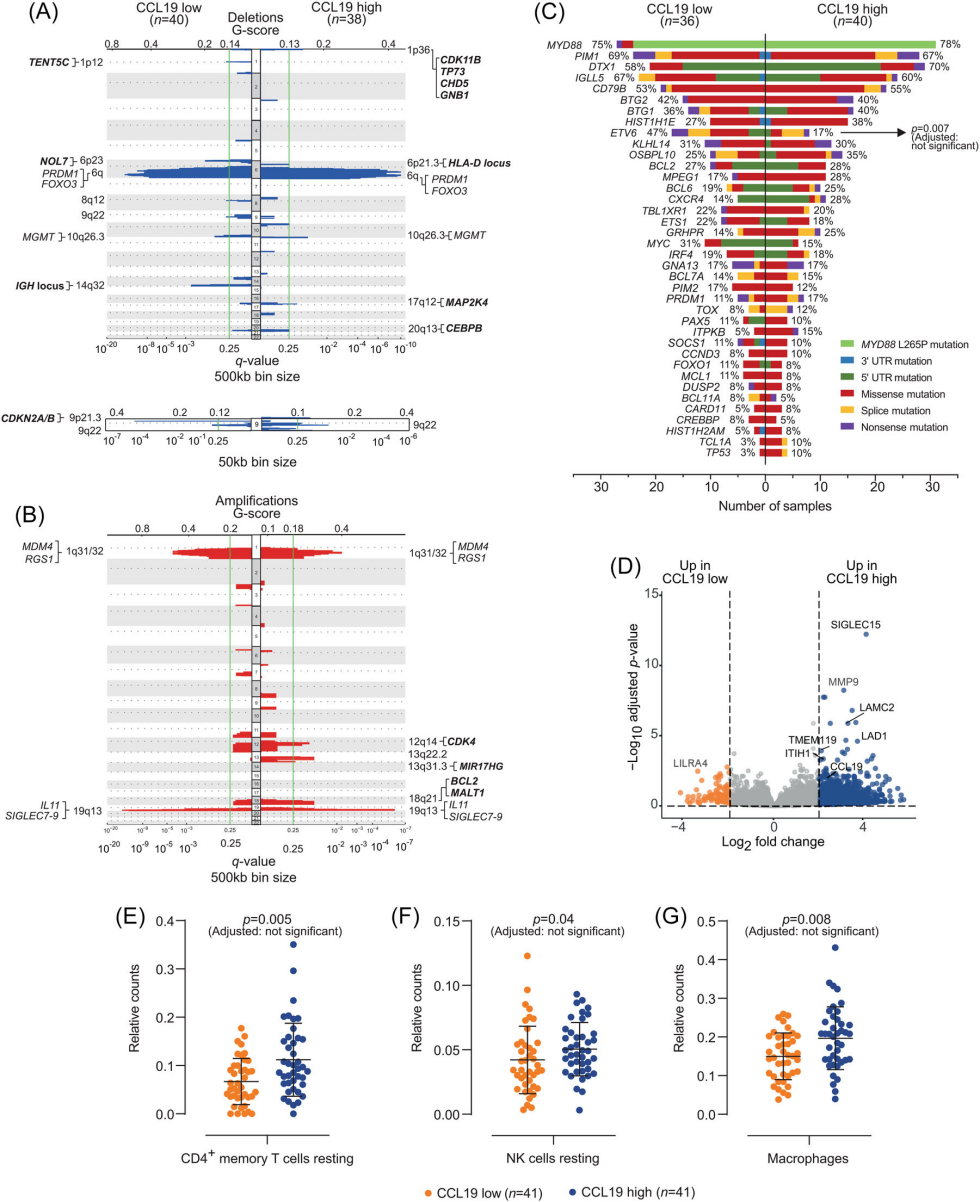
**Association of CCL19 expression with genomic and transcriptomic signatures**. Copy number gains **(A)** and copy number losses **(B)** in “CCL19 low” and “CCL19 high” tumors, shown in GISTIC plots for each subgroup. Significantly altered regions/peaks are annotated using the respective cytoband. Selected candidate genes in these regions/peaks are stated and highlighted in bold when they are located within regions affected by significant copy number variations (CNVs) that are unique to one subgroup. The analysis was performed using a 500 kb bin size, except for chromosome 9. Here, we applied a 50 kb bin size to identify small losses that involve *CDKN2A/B*. Significance was calculated using the GISTIC 2.0 permutation test with Benjamini–Hochberg correction for multiple testing. Green lines represent the significance thresholds. **(C)** Mirrored bar graph comparing the mutational profiles of “CCL19 low” and “CCL19 high” tumors, ordered by their frequency. Mutation types are color‐coded (see legend). The fraction of patients within a specific subgroup carrying a mutation is given next to each bar. There were no significant differences of mutational profiles between the subgroups after applying Benjamini–Hochberg correction for multiple hypothesis testing (threshold: 0.0013). UTR, untranslated region. **(D)** Volcano plot showing differentially expressed genes of samples in the “CLL19 high” (blue) versus “CCL19 low” (orange) group. The Log_2_ fold change cut‐off was set to 2. Selected genes are highlighted. **(E–G)** Dot plots comparing relative counts of three distinct cell populations quantified by CIBERSORTx in “CCL19 high” and “CCL19 low” samples. Adjusted P‐value threshold for multiple testing was set at 0.003 (Benjamini–Hochberg). Bold line, median; error bars, 95% confidence interval.

**Figure 3:**
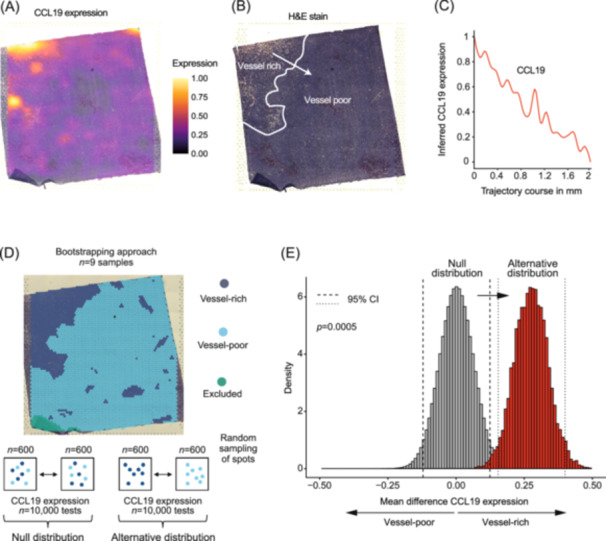
**Spatial distribution of CCL19 in central nervous system lymphoma (CNSL)**. **(A)** Projection of CCL19 expression in a representative tumor slice by spatial transcriptomics. **(B)** Hematoxylin and eosin (H&E) stain of the same tumor slice used for spatial transcriptomics. The white line represents the border between “vessel‐rich” and “vessel‐poor” areas of the tumor, and the white arrow highlights a spatial trajectory from the “vessel‐rich” to “vessel‐poor” area. **(C)** Line plot of inferred CCL19 expression over the course of the trajectory (see B). **(D)** Schematic overview of the image segmentation and bootstrapping analysis. Dots located in “vessel‐rich” regions are colored in dark blue, those in “vessel‐poor” areas are colored in light blue. **(E)** Density plot showing the null (gray) and alternative distribution (red) of the mean differences in CCL19 expression, highlighting the enrichment in CCL19 expression in “vessel‐rich” areas. CI, confidence interval.

Based on the CCL19 transcript levels, we stratified CNSL patients into two groups using the median cut‐off (“CCL19 high” vs. “CCL19 low”), with tumors expressing no CCL19 being allocated to the “CCL19 low” group (Figure 1D, Supporting Information S2: Table 1). Importantly, key preanalytical and methodological variables were balanced between these two groups, including tumor material, tissue fixation type, sequencing depth, and pre‐operative corticosteroid exposure (Supporting Information S1: Figure 1). Moreover, we observed no significant differences in clinicopathological or radiological features such as MSKCC/IELSG risk scores, lymphoma localization, cell‐of‐origin (COO), or Epstein‐Barr virus status (Supporting Information S2: Table 1, Supporting Information S1: Figures 2 and 3). Yet, CNSL patients with high CCL19 expression were significantly younger than those with low CCL19 levels (median age: 64 vs. 71 years, respectively, Figure 1E, Supporting Information S2: Table 1).^5^

**Figure 4:**
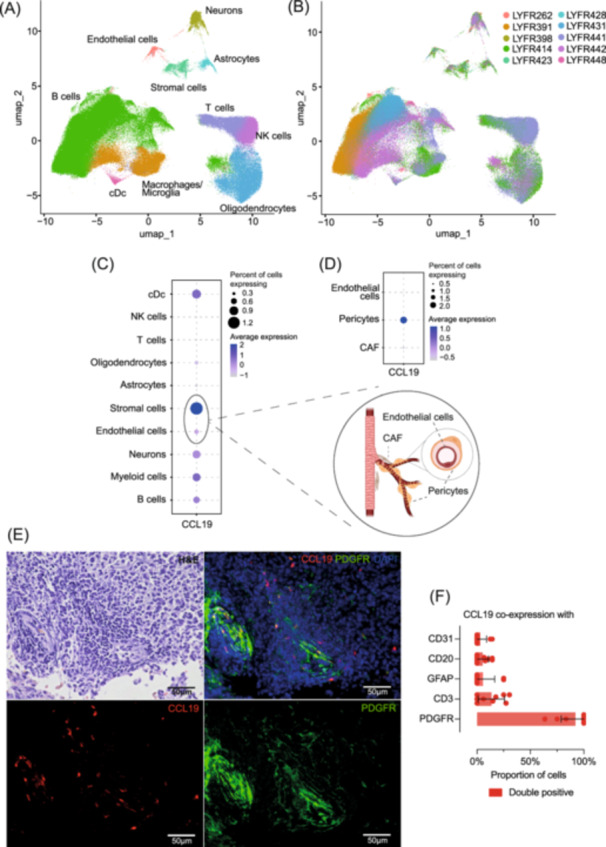
**Cellular origin of CCL19 in central nervous system lymphoma (CNSL)**. **(A)** Uniform Manifold Approximation and Projection (UMAP) plot of 205,000 nuclei from 10 different CNSL tumor samples profiled by single‐nucleus RNA sequencing. Each cluster is color‐coded and annotated with the corresponding cell type. cDC, conventional dendritic cells. **(B)** UMAP plot displaying the same clusters, color‐coded by tumor sample. **(C)** Dot plot showing the expression of CCL19 in the major cell types and a schematic image of the vascular compartment. **(D)** Dot plot demonstrating the expression of CCL19 in the cell types representing the vascular compartment. CAF, cancer‐associated fibroblasts. **(E)** Immunohistochemistry of tumor slices from case LYFR151, showing hematoxylin and eosin (H&E) staining (top left), an overlay of CCL19 (red), the pericyte marker PDGFR (Platelet‐Derived Growth Factor Receptor, green), and nuclei (DAPI, blue) (top right), CCL19 alone (red, bottom left), and PDGFR alone (green, bottom right). Importantly, co‐expression of CLL19 and PDGFR is indicated by the color yellow (top right). **(F)** Bar plot showing the average of the proportion of double‐positive cells among CCL19^+^ cells in all analyzed regions of interest (Supporting Information S1: Methods) for selected cell type‐specific markers, including CD31 (endothelial cells), CD20 (B cells), GFAP (astrocytes), CD3 (T cells), and PDGFR (pericytes).

**Figure 5:**
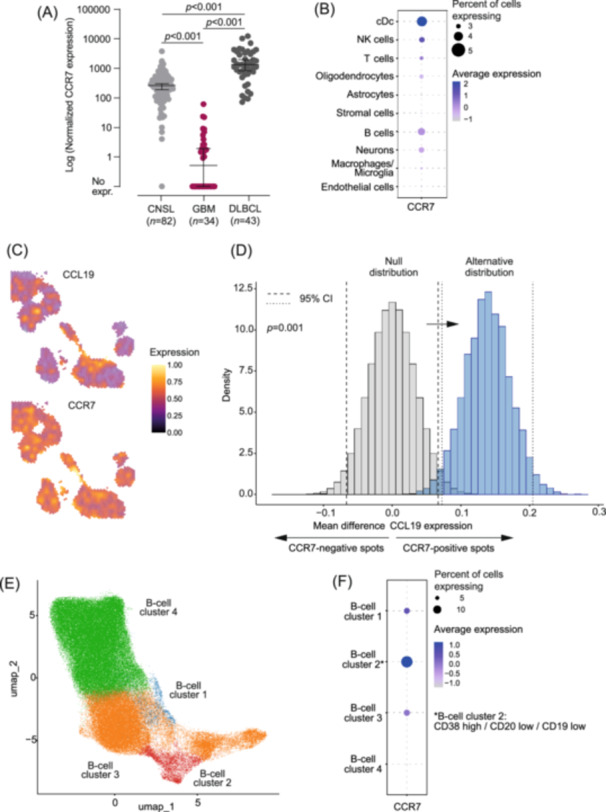
**Co‐localization of CCL19 and CCR7 in central nervous system lymphoma (CNSL)**. **(A)** Comparison of CCR7 expression levels between CNSL, glioblastoma, and systemic diffuse large B‐cell lymphoma. Bold lines represent the median, and error bars show the 95% confidence intervals. DLBCL, diffuse large B‐cell lymphoma; expr., expression; GBM, glioblastoma. **(B)** Dot plot showing the expression of CCR7 within the major cell types identified by single‐nucleus RNA sequencing. cDC, conventional dendritic cells. **(C)** Representative image showing the projection of CCL19 and CCR7 expression on the same tumor slice by spatial transcriptomics. **(D)** Density plot showing the null (gray) and alternative distribution (blue) of the mean differences in CCL19 expression, highlighting the enrichment in CCL19 expression in CCR7‐positive spots “vessel‐rich” areas. CI, confidence interval. **(E)** Uniform Manifold Approximation and Projection (UMAP) plot of the B‐cell subclusters derived from the integrated single‐nucleus RNA sequencing dataset. **(F)** Dot plot showing the expression of CCR7 in these B‐cell subclusters.

Next, we explored the prognostic impact of the CCL19 transcript abundance in CNSL patients undergoing curative‐intent immunochemotherapy (87% of patients, *n* = 71, Figure 1D, Supporting Information S2: Tables 2 and 3). In log‐rank analyses, patients with high CCL19 levels showed significantly inferior PFS (P = 0.008, HR: 2.49, 95% CI: 1.3–4.9) and OS (P = 0.04, HR: 2.43, 95% CI: 1.0–5.3) (Figure 1F,G). Furthermore, in multivariate Cox regression analyses incorporating known prognostic clinical and radiographic risk factors, higher CCL19 levels were significantly and independently associated with unfavorable PFS (P = 0.006) and OS (P = 0.03) (Figure 1H,I). Of note, these survival differences were even more pronounced in the subgroup of CNSL patients that completed consolidation therapy (*n* = 46), with corresponding HRs of 5.1 for PFS (95% CI: 2.1–12.5, P = 0.0003) and 4.9 for OS (95% CI: 1.8–19.8, P = 0.002, Supporting Information S1: Figure 4). While 79% of “CCL19 high” cases in this sub‐cohort experienced lymphoma progression or death within 3 years, 82% of CNSL patients with low CCL19 expression remained disease‐free at the same landmark (Supporting Information S1: Figure 4). In line with these observations, stratification of patients into tertiles according to CCL19 expression revealed particularly poor outcomes among those with the highest CCL19 levels, further supporting a graded association between CCL19 abundance and adverse prognosis (Supporting Information S1: Figure 5). In contrast, we did not observe a prognostic effect of CCL19 in systemic DLBCL (TCGA cohort; PFS, P = *n.s*., HR: 1.0, 95% CI: 0.3–3.1; OS, P = *n.s*., HR: 2.49, 95% CI: 0.3–4.6, data not displayed).

### Association of CCL19 with molecular profiles and immune cell landscapes

We next investigated whether tumors classified as “CCL19 high” or “CCL19 low” exhibited distinct genetic/transcriptomic profiles or immune cell landscapes within their LME. While both groups shared common copy number variations (CNVs) assessed by sWGS, such as deletions of 6q (*PRDM1*, *FOXO3*), 9q22, or 10q26.3 (*MGMT*), and gains of 1q31/32 (*MDM4*, *RGS1*) or 19q13 (*IL11*, *SIGLEC7‐9*), we also observed a substantial number of aberrations that were unique to either cohort (Figure 2A,B, Supporting Information S1: Figure 6, and Supporting Information S2: Table 4). “CCL19 high” tumors showed an enrichment of CNVs affecting genes implicated in immune response (del(6p21.3), *HLA* locus) and apoptotic control (del(1p36), *TP73* and gain(18q21), *BCL2*), suggesting impaired anti‐lymphoma immune activity and potential resistance to apoptosis within this subgroup (Figure 2A,B). On the other hand, cell cycle control appeared dysregulated in both groups, with *CDKN2A/B* (9p21.3) being more frequently deleted in the “CCL19 low” cohort, while “CCL19 high” cases revealed higher rates of *CDK4* amplifications (12q14) (Figure 2A,B).

Targeted capture sequencing by CAPP‐Seq displayed a general mutational profile specific to CNSL, with *MYD88*, *PIM1*, *DTX1*, *CD79B*, and *BTG1* being among the most frequently altered genes across the cohort (Figure 2C, Supporting Information S1: Figure 7A, and Supporting Information S2: Table 5). Most tissue samples were classified as MCD subtype by LymphGen (49%), while 32% of samples were defined as “Others,” 5% as BN2, and 1% as ST2 or EZB (Supporting Information S1: Figure 7B). The MCD subgroup did not represent an unfavorable risk factor in our cohort, in contrast to systemic DLBCL (Supporting Information S1: Figure 7C). Notably, gene‐level mutational patterns, LymphGen subtypes, as well as B‐cell states and lymphoma ecotypes were well balanced between the “CCL19 high” and “CCL19 low” subgroups, exhibiting no significant differences (Figure 2C, Supporting Information S1: Figures 7B and 8).

We next applied RNA sequencing to dissect differentially expressed genes and define the immune cell composition within tumor specimens. CNSL cases with high CCL19 expression displayed upregulation of several genes involved in immune response (*SIGLEC15*, *TMEM119*, and *LILRA4*) and extracellular matrix remodeling (*MMP9*, *ITIH1*, and *LAD1*) (Figure 2D, Supporting Information S2: Table 6). For example, the most abundant transcript *SIGLEC15*, encoding for a protein expressed by myeloid cells and known to suppress T‐cell activity, was significantly upregulated, suggesting an LME skewed towards immune suppression. Further, gene set enrichment analysis revealed an enrichment of apoptosis‐associated genes and genes involved in antigen processing and presentation in the “CCL19 low” subgroup (Supporting Information S1: Figure 9). Finally, deconvolution of cellular subsets by CIBERSORTx uncovered an enrichment of macrophages as well as NK cells and CD4^+^ memory T cells in a resting state in CNSL samples with high CCL19 levels (Figure 2E–G, Supporting Information S1: Figures 10 and 11, *n.s*. after correction).

Collectively, these results suggest an association of CCL19 with anti‐apoptotic and immunosuppressive modifications of the lymphoma cells and the LME, potentially mediating resistance against cytotoxic therapy and contributing to unfavorable clinical outcomes.

### Pericytes constitute the primary source of CCL19 in CNSL

Having defined the clinical relevance of CCL19 in CNSL, we next explored its spatial and cellular distribution within the unique brain LME, profiling nine tumor specimens by the 10x Visium platform (Figure 1A, Supporting Information S1: Figure 12). In all cases, CCL19 expression was enriched in certain spots and areas of the tumor specimens (Figure 3A, Supporting Information S1: Figure 12). By comparing these spatial CCL19 expression patterns with the original H&E‐stained images, CCL19 appeared to be particularly present in vascularized regions of the lymphoma sections (Figure 3B, Supporting Information S1: Figure 12). This observation was supported by a substantial decline in CCL19 expression along trajectories extending from vessel‐rich to vessel‐poor regions within the tumor tissue (Figure 3B,C). In addition, this independent annotation was validated in gene set enrichment analyses, demonstrating a significant overexpression of genes involved in vascular processes within vessel‐rich areas of the tumor tissue (Supporting Information S1: Figure 12). To systematically quantify this effect, all nine H&E‐images were independently annotated by a neuropathologist to distinguish vascularized from non‐vascularized tissue compartments (Figure 3D, Supporting Information S1: Figure 13, and Supporting Information S1). We then aggregated the spots of the spatial images that corresponded to these annotated regions (“vessel‐rich” vs. “vessel‐poor”) across all nine tumor specimens (Figure 3D). Using this combined dataset, we applied a bootstrapping approach to assess differences in CCL19 expression levels between vascularized and non‐vascularized loci and observed a significant enrichment of CCL19 in spots that align with vessel‐rich areas of the CNSL specimens (P = 0.0005, Figure 3D,E).

In a separate analysis, we applied single‐nucleus RNA sequencing to nuclei suspensions from ten different CNSL samples to delineate the cellular origins of CCL19 (Figure 1A, Supporting Information S2: Table 7). From a dataset of 205,000 nuclei, we identified ten distinct cell types, mirroring the expected cellular composition and heterogeneity of the brain LME (Figure 4A, Supporting Information S1: Figure 14A). While B cells comprised the predominant population in the majority of samples, the full cellular repertoire was preserved in all but two cases (Figure 4B, Supporting Information S1: Figure 14B). Within the 10 cell clusters, we observed that CCL19 expression was most abundant in the stromal cell population (Figure 4C). This finding, together with the results of the spatial transcriptomic analyses, led us to focus on the vascular compartment of the tumor microenvironment, which includes stromal and endothelial clusters composed of pericytes, cancer‐associated fibroblasts, and endothelial cells (Figure 4C, Supporting Information S1: Figure 15, and Supporting Information S2: Table 8). Within these subpopulations, CCL19 was almost exclusively expressed in pericytes, a cell type that surrounds small vessels and capillaries in the brain and represents a key component of the blood‐brain barrier (Figure 4D).^22^ We further validated these results at the proteomic level by immunohistochemistry in four cases, demonstrating predominant co‐localization of CLL19 with the pericyte marker PDGFR (Figure 4E, Supporting Information S1: Figures 16 and 17). Specifically, on average 92% of all CCL19^+^ cells co‐expressed PDGFR across all analyzed cases and regions of interest (Figure 4F). In contrast, CCL19 expression was largely absent in other cell types defined by the respective canonical markers including B cells (CD20), T cells (CD3), astrocytes (GFAP), and endothelial cells (CD31) (Figure 4F, Supporting Information S1: Figures 17 and 18). Notably, CCL19‐expressing cells were predominantly surrounded by malignant B cells; yet, T lymphocytes were also detected within those regions, suggesting that these areas could represent tertiary lymphoid structures (Supporting Information S1: Figures 17 and 18).

Collectively, these results identify pericytes within vascularized areas of the brain LME as the primary source of CCL19 in CNSL patients.

### Co‐localization of CCL19 and CCR7 in CNSL

In the next step, we evaluated the role of CCR7, the primary chemokine receptor of CCL19, in human CNSL samples, examining its cellular distribution and spatial interactions with CCL19. CCR7 transcripts were highly abundant in CNSL, with expression levels significantly exceeding those in GBM (P = 3.3e−16), but remaining lower than in systemic DLBCL (P = 7e−10 TCGA cohort, Figure 5A). Dendritic cells (DCs), NK cells, T cells, and B cells exhibited the highest CCR7 expression levels among all cell types, consistent with previous reports in systemic lymphomas and healthy conditions, as well as with an independent cohort of PCNSL samples (Figure 5B, Supporting Information S1: Figure 19).^23^, ^24^, ^25^, ^26^, ^27^, ^28^, ^29^ In individual tumor slices, we observed a prominent co‐localization of CCR7 and its ligand CCL19 by spatial transcriptomics analyses (Figure 5C). This relationship was further confirmed by a significant enrichment of CCL19 expression in CCR7‐positive spots within the integrated dataset from all eight CNSL samples (P = 0.001, Figure 5D).

To further investigate CCR7 in these specific cell compartments, we performed subclustering of the B‐cell and NK‐/T‐cell populations based on our integrated single‐nucleus RNA sequencing dataset of ten CNSL specimens (Figure 5E, Supporting Information S1: Figures 20 and 21). We identified four distinct B‐cell and four NK‐/T‐cell subclusters, which displayed cell type‐specific transcriptomic profiles (Figure 5E, Supporting Information S1: Figures 20 and 21). Within these cell populations, CCR7 was predominantly expressed in DCs, NK cells, CD4^+^ T cells, and plasmablast‐like B cells, which were characterized by CD38‐positivity and have been associated with poor clinical outcomes in previous studies (Figure 5F, Supporting Information S1: Figure 22).^30^

## DISCUSSION

We here provide a comprehensive characterization of CCL19 and its biological and clinical relevance in patients with brain lymphomas. Leveraging an integrated multi‐omics framework including spatial and single‐cell technologies, we analyzed 82 tumor specimens and identified pericytes in vessel‐rich areas as the predominant source of CCL19 expression. In these regions, CCL19 co‐localized with its primary receptor CCR7, which was mainly expressed by plasmablast‐like malignant B cells, as well as DCs, NK cells, and CD4^+^ T cells. High CCL19 expression was strongly associated with unfavorable clinical outcomes, as demonstrated by both log‐rank and Cox regression analyses. Notably, the prognostic impact was even more evident in patients who completed consolidation therapy, suggesting a robust biological effect that is unlikely to be confounded by treatment‐related morbidity and mortality. Furthermore, tumor samples with high CCL19 levels revealed distinct copy number and transcriptomic signatures as well as cellular compositions indicative of impaired immunosurveillance and apoptotic regulation. These included higher frequencies of *TP73* deletions and *BCL2* gains, an increased rate of *HLA* losses, upregulation of genes involved in immunosuppression such as *SIGLEC15*, and elevated numbers of resting T and NK cells. Notably, the somatic mutation landscape remained balanced between “CCL19 high” and “CCL19 low” tumors, underscoring the remarkably homogeneous mutation profile characteristic of this disease.

Under physiological conditions, chemokines are crucial regulators of cell migration, immune tolerance, and inflammatory responses.^31^ CCL19 and its primary receptor CCR7 orchestrate the directed migration of immune cells into secondary lymphoid organs, where they mediate essential processes including antigen surveillance, tolerance, and adaptive immune responses. Physiologically, CCR7 is expressed in naïve and memory T cells, regulatory T cells, B cells, and DCs, guiding their trafficking into lymphoid structures along CCL19 and CCL21 gradients, which are mainly expressed by fibroblastic reticular cells of the stromal cell compartment.^31^, ^32^ The CCR19–CCL7 axis has been implicated in the metastatic dissemination of various solid tumors, and their interaction has been shown to be critical for CNS infiltration in murine T‐cell leukemia models.^33^, ^34^ In systemic DLBCL and transformed follicular lymphoma patients, high CCR7 and CCL19 expression levels were observed, with CCR7 abundance being associated with unfavorable prognosis.^25^ In addition, it has been demonstrated in murine B‐cell lymphoma models that CCR7 mediates homing of the tumor cells into lymphoma‐supporting niches of secondary lymphoid organs, suggesting that autocrine CCL19–CCR7 signaling might contribute to lymphomagenesis in aggressive B‐cell lymphomas.^35^

Little is known about the impact of CCL19 and CCR7 in patients with CNSL. Two studies analyzing human tumor specimens have reported that CCR7 expression is readily detectable in both primary and SCNSL.^36^, ^37^ A seminal research study in murine CNSL models showed stimulation of CCL19 production in astrocytes of the brain through NF‐κB activation, leading to parenchymal retention of lymphoma cells. Furthermore, age‐related and NF‐κB‐induced gliosis increased the risk of forming CNSL in vivo, suggesting a mechanism driving CNS pathogenesis and tropism.^5^ Motivated by these findings in mice, we comprehensively and deeply characterized the origins and prognostic impact of CCL19 in human CNSL tumor specimens in this study. We found that CCL19 and CCR7 were readily detectable in CNSL, albeit at lower levels compared to systemic aggressive B‐cell lymphomas, mirroring findings from previous work in DLBCL.^25^ While CCL19 expression could be clearly attributed to pericytes within the stromal compartment of vascularized tumor regions, CCR7 was expressed by various cell types, predominantly by malignant B cell, T cells, NK cells, and DCs. We further observed a trend for correlation between CCL19 expression levels and CNSL tumor burden, as well as spatial co‐localization of CCL19 and CCR7, reinforcing prior evidence that CCL19 and the interaction with its receptor might facilitate the retention of malignant B cells within the brain parenchyma and promote their migration into supportive microenvironmental niches. This mechanism and the localization of CCL19 within the stromal compartment of the brain potentially mirror its established physiological role in guiding lymphocyte positioning within lymphoid organs and its proposed function in systemic B‐cell lymphomas. Therefore, our results, alongside the findings in murine models, support a role for CCL19 and the CCL19–CCR7 axis in CNS lymphomagenesis and tropism, potentially orchestrating a lymphoid‐like architecture within the CNS via tertiary lymphoid structures, which have been described in other neuropathological conditions such as GBM and autoimmune diseases including multiple sclerosis.^38^, ^39^, ^40^, ^41^ Notably, several clinical case reports indicate that chronic inflammatory brain diseases might precede the onset of CNSL; yet, a definitive link between neuroinflammation and CNSL development remains unproven.^42^, ^43^, ^44^, ^45^, ^46^ While this mechanism represents only one potential model for the complex biology underlying CNSL tropism, additional processes are likely involved in governing lymphoma cell migration, including antigen recognition and chronic antigenic stimulation of the B‐cell receptor pathway through CNS‐specific proteins.

We here demonstrated for the first time that CCL19 represents an adverse prognostic marker in CNSL, associated with tumor‐intrinsic and LME‐related features contributing to brain lymphoma proliferation and survival, including impaired immunosurveillance, enhanced anti‐apoptotic signatures, and a cold immune microenvironment. While our results do not allow conclusions towards biological causality, these findings might provide a rationale to explore therapeutic targeting of the CCL19–CCR7 axis. Two CCR7‐directed agents, the anti‐CCR7 antibody CAP‐100 (NCT04704323) and the antibody–drug conjugate JBH492 (NCT04240704), are currently investigated in Phase I trials for systemic non‐Hodgkin lymphomas. However, patients with CNS involvement are excluded from these studies, underscoring the need for dedicated evaluation of these therapeutic strategies in CNSL.

Collectively, our study identifies pericyte‐derived CCL19 in vascularized regions as an adverse prognostic factor in CNSL, with a significant CCR7 spatial co‐localization and association with unfavorable outcomes, higher tumor burden, as well as anti‐apoptotic and immunosuppressive signatures in malignant B cells and the surrounding microenvironment. These results suggest CCL19 and the CCL19–CCR7 axis as important factors for CNS lymphomagenesis and survival, defining this mechanism as a potential therapeutic target in brain lymphoma patients.

## AUTHOR CONTRIBUTIONS

**Julia C. Kuehn**: Conceptualization; methodology; data curation; investigation; formal analysis; funding acquisition; visualization; project administration; writing—original draft; writing—review and editing. **Lauritz Miarka**: Data curation; formal analysis; methodology; writing—review and editing. **Roman Sankowski**: Methodology; formal analysis; resources; writing—review and editing. **Jurik Mutter**: Data curation; formal analysis; writing—review and editing. **Nicolas N. Neidert**: Formal analysis; resources; writing—review and editing. **Elena Grabis**: Methodology; data curation; writing—review and editing. **Junyi Zhang**: Methodology; writing—review and editing. **Christian Klingler**: Formal analysis; writing—review and editing. **Fabian Hummel**: Methodology; data curation; formal analysis; writing—review and editing. **Lavanya Ranganathan**: Formal analysis; writing—review and editing. **Sabine Bleul**: Project administration; resources; writing—review and editing. **Eliza M. Lauer**: Data curation; investigation; writing—review and editing. **Dieter H. Heiland**: Methodology; supervision; writing—review and editing. **Katharina Müller**: Project administration; writing—review and editing; resources. **Hans C. Reinhardt**: Supervision; writing—review and editing. **Sascha Dietrich**: Supervision; writing—review and editing. **Gerald Illerhaus**: Investigation; supervision; writing—review and editing. **Louisa von Baumgarten**: Resources; writing—review and editing. **Stefan Alig**: Formal analysis; writing—review and editing. **Maximilian Diehn**: Methodology; supervision; writing—review and editing. **Bastian E. A. Sajonz**: Resources; writing—review and editing. **Jürgen Beck**: Resources; writing—review and editing. **Volker A. Coenen**: Resources; writing—review and editing. **Marco Prinz**: Supervision; resources; writing—review and editing. **Elisabeth Schorb**: Supervision; writing—review and editing; Resources. **Ash A. Alizadeh**: Methodology; supervision; writing—review and editing. **Justus Duyster**: Supervision; resources; writing—review and editing. **Peter Reinacher**: Supervision; resources; writing—review and editing. **Florian Scherer**: Conceptualization; methodology; formal analysis; validation; funding acquisition; visualization; project administration; writing—original draft; supervision; investigation; writing—review and editing; data curation; resources.

## CONFLICT OF INTEREST STATEMENT

F.S. receives research funding from Gilead Sciences, Roche Sequencing Solutions, and Takeda, and received honoraria from AstraZeneca and Servier. H.C.R. received consulting and lecture fees from AbbVie, Roche, KinSea, Vitis, Cerus, Lilly, Novartis, Takeda, AstraZeneca, Vertex, and Merck. H.C.R. received research funding from AstraZeneca and Gilead Pharmaceuticals. H.C.R. is a co‐founder of CDL Therapeutics GmbH. N.N.N. served on advisory boards for Servier and serves as a consultant for B. Braun New Ventures. B.E.A.S. serves as an advisor for Precisis (Heidelberg, Germany) and receives a research grant from Ceregate (Hamburg, Germany) and has received travel support from Boston Scientific (Marlborough, MA, USA). For the remaining authors, no relevant conflict of interest was declared.

## FUNDING

This work was supported by the Berta‐Ottenstein Programm Förderlinie Clinician Scientist (to J.C.K. and L.M.), the Advanced Clinician Scientist Program of the Deutsche Gesellschaft für Innere Medizin (to F.S.), the Deutsche Forschungsgemeinschaft (SCHE 1870/3‐1, to F.S.), the Else Kröner‐Fresenius‐Stiftung (2018_A38, to F.S.) and Exzellenzstipendium of the Else Kröner‐Fresenius‐Stiftung (to F.S.), the Mertelsmann Foundation (to F.S.), the Fördergesellschaft Forschung Tumorbiologie (to F.S.), and the German Cancer Consortium (DKTK, to F.S.). R.S. is supported by the IMMediate Advanced Clinician Scientist‐Program, Department of Medicine II, Medical Center – University of Freiburg and Faculty of Medicine, University of Freiburg, funded by the Bundesministerium für Bildung und Forschung (BMBF, Federal Ministry of Education and Research) – 01EO2103. Furthermore, R.S. is supported by the Fritz Thyssen Foundation and the Deutsche Forschungsgemeinschaft (SFB‐1479 – Project ID: 441891347). Open Access funding enabled and organized by Projekt DEAL.

## Supporting information

Supporting Information.

Supporting Information.

## Data Availability

The data that support the findings of this study are available on request from the corresponding author. The data are not publicly available due to privacy or ethical restrictions. Tumor mutational and transcription data and other relevant data are provided in the Data Supplement. Owing to restrictions related to the dissemination of germline sequence information included in the informed consent forms used to enroll study subjects, we are unable to provide access to raw sequencing data. Reasonable requests for additional data will be reviewed by the authors to determine whether they can be fulfilled in accordance with these privacy restrictions.
