# Supplementary material for: Spatial and multi‐omic profiling reveals pericyte‐derived CCL19 as a key prognostic factor in CNS lymphoma
Source: Hemasphere. 2026 Jun 22;10(6):e70412. doi: 10.1002/hem3.70412 (PMC13285587; doi:10.1002/hem3.70412)
Supplement: Supplementary file 1 — Supporting Information. [file HEM3-10-e70412-s002.pdf]

Supporting Information for

Spatial and multi-omic profiling reveals pericyte-derived CCL19 as a key prognostic factor in CNS lymphoma

Table of contents

Supporting Information: Methods

Supporting Information: References

Supporting Information: Figures

Supporting Information: Tables – attached separately

Table 1. Clinicopathological characteristics of the entire cohort.

Table 2. Clinicopathological characteristics of patients undergoing curative-intent therapy.

Table 3. Case-level patient characteristics.

Table 4. Detected CNAs in the entire cohort.

Table 5. Detected SNVs in the entire cohort.

Table 6. Differentially expressed genes in 'CCL19 high' vs. 'CCL19 low' subgroups.

Table 7. Number of nuclei analyzed per sample.

Table 8. List of genes used to define stromal cell subpopulations.

Supporting Information: Methods

Patient enrollment, sample and data collection

Of the 92 patients, 77 have been enrolled and treated at the Department of Hematology and Oncology, the Department of Stereotactic and Functional Neurosurgery as well as the Department of Neurosurgery of the University Medical Center Freiburg, Germany. An additional cohort of 15 patients was enrolled and treated at the Department of Neurology, University Hospital, LMU Munich. From all patients, blood samples were available that were used to obtain DNA as germline controls (see below). Of the 82 CNS lymphoma (CNSL) specimens, 74 were obtained by stereotactic biopsies, while 8 patients underwent resection due to suspected glioma/brain metastases (**Supporting Information: Table 3**). All tumor samples were obtained prior to initiation of CNSL-specific therapy. Tumor tissue was either snap frozen and stored in liquid nitrogen up to 6 months or tissue was fixated with formalin and embedded in paraffin according to clinical routine procedures.

Measurement of MRI tumor volumes

For 61 patients, MRI scans at CNSL diagnosis were available, acquired on 1.5 or 3.0 Tesla scanners as 3D volumetric acquisitions conducted as part of routine clinical management. 3D tumor volumes were quantified using semi-automated segmentation of contrast-enhanced T1w images by the software module Smartbrush of Elements® (Brainlab, Munich, Germany), as described previously [1]. Tumor volumes were quantified in mL. Other radiographic features such as deep brain involvement, number of tumor lesions, periventricular involvement, infratentorial involvement, bi-hemispheric

involvement, meningeal involvement as well as ependymal involvement were extracted from radiological reports if information was available.

DNA and RNA isolation

DNA and RNA were isolated from up to five 10 μm sections of FFPE bulk tumor tissue or available fresh frozen tissue using the AllPrep DNA/RNA FFPE Kit (QIAGEN, Venlo, Netherlands) according to manufacturer's instructions. Tumor DNA yield was in median 393 ng and ranged from 6.9 to 7,380 ng. Tumor RNA yield was in median 1,038 ng and ranged from 0.9 to 70,200 ng. DNA from 200 μl of plasma-depleted whole blood was used as germline controls. Plasma-depleted whole blood was obtained from all patients by centrifugation of blood collected in EDTA tubes at 800 x g for 10 minutes as described before [2]. DNA from plasma-depleted whole blood was isolated using the QIAamp DNA mini kit (QIAGEN) according to manufacturer's instructions. The genomic DNA isolated from tumor and plasma-depleted whole blood was further sonicated using the Covaris system, as described previously [2]. RNA quality was assessed using High Sensitivity RNA ScreenTape Analysis assay on a Tape Station (Agilent, Santa Clara, CA, USA). Samples with a DV200 above 50% were used for further bulk RNA sequencing.

Tumor bulk RNA sequencing

Ribosomal RNA was depleted using the NEBNext rRNA Depletion Kit v2 (New England Biolabs, Ipswich, MA, USA). Library preparation was performed using the NEBNext Ultra II Directional RNA Library Prep Kit according to manufacturer's instructions. 51 libraries were sequenced using a P2 Flow Cell (100 cycles) on the Illumina NextSeq1000 platform

and 41 libraries were sequenced using a High output Flow cell (75 cycles) on the Illumina NextSeq550 platform (Illumina, San Diego, CA, USA). Data were processed as previously described [3]. Briefly, fastq files were trimmed using Cutadaptor and reads were aligned to the reference genome with STAR [4,5]. Count data were generated using featureCounts, followed by normalization utilizing the DESeq2 tool, a widely used method that models count data with a negative binomial distribution and applies sample-specific size factors to correct for differences in sequencing depth and RNA composition [6,7]. Differentially expressed genes were identified by the Deseq2 standard workflow [7]. Gene set enrichment analysis were performed and visualized using the fgsea package workflow[8]. The ecotypes and cell states of the lymphoma samples were assessed using the Lymphoma EcoTyper [9]. Furthermore, RNA sequencing data from 42 DLBCL patients of The Cancer Genome Atlas (TCGA) Program (dbGaP accession: phs000178) and from 24 glioblastoma cases from the Ivy Glioblastoma Atlas Project were available which were normalized in the same fashion for comparison of gene expression levels [10].

Deconvolution with CIBERSORTx

To estimate the cellular composition based on bulk gene expression data, the deconvolution tool CIBERSORTx was applied [11]. To estimate the immune cell composition, the LM22 signature matrix was used; for the estimation of CNS-specific cell populations, a single-cell dataset of 2 human healthy brain samples (Human M1 10x dataset) was utilized (10, Allen Institute for Brain Science. Available from: https://portal.brain-map.org/atlases-and-data/rnaseq/human-m1-10x). In the healthy brain single-cell dataset, various neuronal subtypes were originally annotated separately. For

the purposes of generating a simplified CNS cell-type signature matrix, all neuronal subtypes were merged into a single category labeled ‘neurons’, without further subclassification. Furthermore, we used CIBERSORTx for the quantification of plasmablast-like B cells. Here, we generated a signature matrix based on the single-cell dataset from the annotated B-cell subpopulations of ten CNSL samples (see below). All the different signature matrices were generated using the standard settings. To impute cell fractions, batch correction in B-mode was performed for the microarray-based LM22 signature matrix and S-mode for the single-cell-based signature matrix files. 100 permutations were used for significance analysis.

Cell-of-origin (COO) classification

The COO of CNSL samples was determined by immunohistochemistry according to the Hans classifier (this information was only available in patients diagnosed after June 2020). Further, we assessed activated B-cell like (ABC) and germinal center B-cell like (GCB) scores based on the expression of 9 GCB- and 11 ABC-associated genes as described by Reddy et al. [12]. The GCB/ABC score was calculated averaging the Z-normalized expression values. Samples with a subtype score >0.25 were classified as ABC, those with a score <-0.25 samples were classified as GCB. Samples not fulfilling these criteria were considered unclassified.

Targeted capture sequencing and shallow whole genome sequencing (sWGS)

Cancer Personalized Profiling by Deep Sequencing (CAPP-Seq) was performed for DNA mutational profiling of the CNSL samples, using a CNSL-specific custom sequencing

panel that has been introduced previously [2]. Sequencing was performed on the Illumina NextSeq 1000 and Illumina NextSeq 550 platforms. All steps of the CAPP-Seq workflow have been reported and described in detail previously [2]. For the sWGS analyses, sequencing was performed on the Illumina NovaSeq6000 platform. Genome-wide copy number aberrations analysis was conducted for bin sizes of 50 and 500 kb using ichorCNA [3,13]. ichorCNA was run using the corresponding WIG files, applying default parameters. A Panel of Normals (PoN), generated from 70 germline samples obtained from patients with CNSL, was used to enhance detection accuracy. These germline samples were sequenced in the same fashion as described above. Then, the GISTIC pipeline was used to identify regions of the genome that are significantly amplified or deleted across all analyzed samples [14].

Single-nucleus RNA sequencing

Fresh frozen tissue from ten CNSL patients, stored in liquid nitrogen, was thawed on ice. Pre-cooled EZ Lysis Buffer (Sigma-Aldrich, St. Louis, MO, USA) was added and the tissue was homogenized using a micro-pestle. The homogenate was incubated for 5 min and centrifuged for 5 min at 500 g. The pellet was resuspended in EZ Lysis Buffer and incubated for another 5 min. After centrifugation at 500 g for 4 min, the supernatant was discarded and the nuclei were resuspended in 500 μl pre-cooled Nuclei Suspension Buffer (1% BSA, 0.2 U/μl RNAse inhibitor). The nuclei were then filtered using a 70 μm Flowmi Strainer. After another centrifugation step (500 g, 5 min, 4°C), nuclei were washed with Nuclei Suspension Buffer. After filtering with a 40 μm Flowmi Strainer, nuclei were counted, and quality was visually assessed. After an additional centrifugation step at 300

g for 10 min at 4°C, nuclei were again resuspended and loaded onto a Chromium X Controller (10x Genomics, Pleasanton, CA, USA) using the Next GEM Single Cell 3' Reagent Kit v3.1 and the GEM-X Universal 3' Gene Expression Reagent Kit v4. Library construction and sample indexing was performed according to the manufacturer's instructions. Each library was sequenced to a depth of approximately 20,000 reads per cell using a P2 Flow Cell (100 cycles) on the Illumina NextSeq1000 platform. After sequencing, reads were aligned to the human reference (GRCh38-2020-A), single nuclei were identified, and expression levels of individual genes per nucleus were defined using the CellRanger v.7.2.0 and CellRanger v.8.0 workflow. The resulting filtered counts matrices were further analyzed by the Seurat 5.1 package [15]. Cells with >10% mitochondrial transcripts, >7500 or <200 features per cell were excluded from all subsequent analyses. In addition, doublet removal was performed using the DoubletFinder 2.0 R package [16]. Then, all single-nuclei datasets were integrated using Harmony 1.2 [17]. Dimensionality reduction was performed by computing a Uniform Manifold Approximation and Projection (UMAP) embedding, using the first 30 Harmony-corrected principal components. Next, a shared nearest neighbor (SNN) graph was constructed by these same components to display the local neighborhood structure using FindNeighbors(). Then, clustering was performed on the SNN graph using the Seurat's FindClusters() function, with a resolution parameter set to 0.3. To annotate clusters with putative cell identities, we employed both a reference-based and a data-driven approach. For the reference-based approach, SingleR with the celldex HumanPrimaryCellAtlasData dataset was used to assign labels based on the closest transcriptomic match [18,19]. To further support and refine annotation, we applied the data-driven approach, for which we

identified cluster-specific marker genes using Seurat's FindAllMarkers() function and compared these to canonical lineage markers. Dot plot of expression data was generated with the DotPlot() command, showing the fraction of cells expressing the gene as dot size and the scaled average expression of the gene in the cluster as color scale.

Separately, to further characterize the vascular compartment of the single-nuclei dataset, i.e. the endothelial and stromal cell clusters (**Supporting Information: Figure 14**), we first extracted these clusters from the integrated dataset using Seurat's subset() function. In order to differentiate pericytes from cancer associated fibroblasts within the stromal cell cluster, expression signatures were curated based on previously published markers that allow to distinguish these populations (**Supporting Information: Table 8**) [20,21]. Using the Ucell package, nuclei of the vascular compartment were then annotated based on the expression scores of these signature genes [22].

The B-cell subclusters shown in **Figure 5E** were generated in the same manner. To assess cell cycle states within these B-cell subclusters, we applied Seurat's CellCycleScoring() function that provides a curated set of canonical S-phase and G2/M-phase marker genes. Using Seurat's FindAllMarkers() function, the marker genes for all B-cell subclusters were identified, with high CD38 and low CD19/MS4A1 expression defining the plasmablast-like B-cell cluster.

<u>Spatial transcriptomics</u>

We performed spatial transcriptomics for nine CNSL tumor samples, using two different 10x Visium workflows. First, four FFPE tissue specimens obtained by resection were

analyzed using the Visium Spatial Gene Expression Reagent Kits for FFPE (10x Genomics, Pleasanton, CA, USA), after identifying representative tumor areas based on H&E staining. For this workflow, FFPE tumor blocks were hydrated in an ice bath for 5 to 20 min until the tissue surface developed a glossy appearance. Tissue was cut into 5 μm slices using a microtome with a section transfer system (Fisher scientific, Hampton, NH, USA), then placed in a 40 °C water bath and allowed to float until all wrinkles had disappeared. Slices were then mounted onto capture areas of provided slides and dried on a cycler at 42°C for 1 h and in a desiccator overnight. After these steps, deparaffinization, H&E staining and imaging was performed according to the manufacturer's instructions, applying the Invitrogen EVOS M7000 Imaging System. Library construction was performed according to the manufacturer's instructions. For the second workflow, one resection specimen and four stereotactic biopsies were analyzed using the Visium CytAssist Spatial Gene Expression for FFPE workflow (10x Genomics), following the manufacturer's instructions, which include staining, imaging, tissue section transfer onto provided slides, and library construction.

All generated libraries from both workflows were sequenced on the NextSeq1000 platform (Illumina). Then, sequencing as well as imaging data were processed using the Space Ranger Pipeline v2.1 standard workflow. To further analyze the resulting datasets, we applied the standard workflow of SPATA2 [23]. Expression of CCL19 was assessed using the plotSurface() function.

To perform the bootstrapping analysis and compare vessel-associated gene signatures among annotated compartments shown in **Figure 3D,E** and **Supporting Information:**

Figure 12, all spatial transcriptomics datasets were imported into the Seurat 5.1 package [15]. Segmentation metadata from the SPATA2 processed objects (see Methods in the main manuscript, i.e. the annotated 'vessel-rich' and 'vessel-poor' areas) were extracted and connected to the corresponding Seurat datasets. All datasets from individual slices were then merged and 'SCTransformed'. We extracted gene expression data from the merged SCTransformed integrated spatial dataset using FetchData(). Gene set enrichment was performed using clusterProfiler [24]. Average expression of vascular marker genes was calculated and plotted using the pheatmap package. Next, to define the null distribution, we compared CCL19 expression across 600 randomly selected spots containing equal numbers of spots from 'vessel-rich' and 'vessel-poor' areas ($n = 300$ each) to expression levels in another 600 randomly sampled spots selected in the same fashion. For the alternative distribution, CCL19 expression in 600 randomly sampled spots from 'vessel-rich' areas was compared to 600 randomly sampled spots from 'vessel-poor' areas. For each comparison, the mean difference in CCL19 expression was calculated. This was repeated 10,000 times in a bootstrapping procedure to generate empirical distributions of mean differences under both conditions (i.e., null and alternative). A two-sided Student's t-test was applied to assess the statistical significance of the observed difference between the null and alternative distributions.

To assess co-localization of CCL19 and CCR7 in **Figure 5C,D** a similar approach was applied. Spots with CCR7 expression values >0 were defined as 'CCR7-positive', all others as 'CCR7-negative'. To define the null distribution, we compared the expression of CCL19 across 600 randomly selected spots that contained equal numbers of 'CCR7-positive' and 'CCR7-negative' spots ($n = 300$ each) to expression levels in another 600

randomly sampled spots selected in the same fashion. For the alternative distribution, CCL19 expression in 600 randomly sampled ‘CCR7-positive’ spots was compared to 600 randomly sampled ‘CCR7-negative’ spots. For each comparison, the mean difference in CCL19 expression was calculated. Again, this was repeated 10,000 times in a bootstrapping procedure to generate empirical distributions of mean differences under both conditions (i.e., null and alternative). A two-sided Student’s t-test was applied to assess the statistical significance of the observed difference between the null and alternative distributions.

Extended statistical considerations

For Cox regression analyses displayed in **Figure 1H,I** conventional risk parameters including age were considered as prognostic parameters. Sex was considered as biological variable and compared between the ‘CCL19 high’ and ‘CCL19 low’ subgroups (**Supporting Information: Tables 1,2**). No formal power analysis was performed, as this study involved retrospective analysis of prospectively collected data and was not designed to test a predefined hypothesis. Likewise, blinding was not applicable due to the retrospective nature of the evaluation. Patients considered for outcome analyses were limited to those undergoing curative-intent therapies. **Figure 1D-I** shows patients planned for curative-intent treatment, regardless of whether they fully received induction and consolidation therapy. The outcome analyses for **Supporting Information: Figure 4** were limited to those patients who completed the entire treatment regimen including consolidation therapy.

References

1. Lauer EM, Riegler E, Mutter JA, et al. Improved early outcome prediction by MRI-based 3D tumor volume assessment in patients with CNS lymphomas. *Neuro Oncol*. 2024;26(2):374-386. doi:10.1093/neuonc/noad177

2. Mutter JA, Alig SK, Esfahani MS, et al. Circulating Tumor DNA Profiling for Detection, Risk Stratification, and Classification of Brain Lymphomas. *J Clin Oncol*. 2023;41(9):1684-1694. doi:10.1200/JCO.22.00826

3. Kuehn JC, Neidert NN, Zhang J, et al. The effect of ibrutinib on the myeloid cell compartment in CNS lymphoma. *Leukemia*. 2025;39(6):1532-1535. doi:10.1038/s41375-025-02600-y

4. Martin M. Cutadapt removes adapter sequences from high-throughput sequencing reads. *EMBnet j*. 2011;17(1):10. doi:10.14806/ej.17.1.200

5. Dobin A, Davis CA, Schlesinger F, et al. STAR: ultrafast universal RNA-seq aligner. *Bioinformatics*. 2013;29(1):15-21. doi:10.1093/bioinformatics/bts635

6. Liao Y, Smyth GK, Shi W. featureCounts: an efficient general purpose program for assigning sequence reads to genomic features. *Bioinformatics*. 2014;30(7):923-930. doi:10.1093/bioinformatics/btt656

7. Love MI, Huber W, Anders S. Moderated estimation of fold change and dispersion for RNA-seq data with DESeq2. *Genome Biol*. 2014;15(12):550. doi:10.1186/s13059-014-0550-8

8. Korotkevich G, Sukhov V, Budin N, Shpak B, Artyomov MN, Sergushichev A. Fast gene set enrichment analysis. *Bioinformatics*. Preprint posted online June 20, 2016. doi:10.1101/060012

9. Steen CB, Luca BA, Esfahani MS, et al. The landscape of tumor cell states and ecosystems in diffuse large B cell lymphoma. *Cancer Cell*. 2021;39(10):1422-1437.e10. doi:10.1016/j.ccell.2021.08.011

10. Puchalski RB, Shah N, Miller J, et al. An anatomic transcriptional atlas of human glioblastoma. *Science*. 2018;360(6389):660-663. doi:10.1126/science.aaf2666

11. Newman AM, Steen CB, Liu CL, et al. Determining cell type abundance and expression from bulk tissues with digital cytometry. *Nat Biotechnol*. 2019;37(7):773-782. doi:10.1038/s41587-019-0114-2

12. Reddy A, Zhang J, Davis NS, et al. Genetic and Functional Drivers of Diffuse Large B Cell Lymphoma. *Cell*. 2017;171(2):481-494.e15. doi:10.1016/j.cell.2017.09.027

13. Adalsteinsson VA, Ha G, Freeman SS, et al. Scalable whole-exome sequencing of cell-free DNA reveals high concordance with metastatic tumors. *Nat Commun*. 2017;8(1):1324. doi:10.1038/s41467-017-00965-y

14. Mermel CH, Schumacher SE, Hill B, Meyerson ML, Beroukhim R, Getz G. GISTIC2.0 facilitates sensitive and confident localization of the targets of focal somatic copy-number alteration in human cancers. *Genome Biol*. 2011;12(4):R41. doi:10.1186/gb-2011-12-4-r41

15. Hao Y, Stuart T, Kowalski MH, et al. Dictionary learning for integrative, multimodal and scalable single-cell analysis. *Nat Biotechnol*. 2024;42(2):293-304. doi:10.1038/s41587-023-01767-y

16. McGinnis CS, Murrow LM, Gartner ZJ. DoubletFinder: Doublet Detection in Single-Cell RNA Sequencing Data Using Artificial Nearest Neighbors. *Cell Syst*. 2019;8(4):329-337.e4. doi:10.1016/j.cels.2019.03.003

17. Korsunsky I, Millard N, Fan J, et al. Fast, sensitive and accurate integration of single-cell data with Harmony. *Nat Methods*. 2019;16(12):1289-1296. doi:10.1038/s41592-019-0619-0

18. Aran D, Looney AP, Liu L, et al. Reference-based analysis of lung single-cell sequencing reveals a transitional profibrotic macrophage. *Nat Immunol*. 2019;20(2):163-172. doi:10.1038/s41590-018-0276-y

19. Mabbott NA, Baillie JK, Brown H, Freeman TC, Hume DA. An expression atlas of human primary cells: inference of gene function from coexpression networks. *BMC Genomics*. 2013;14:632. doi:10.1186/1471-2164-14-632

20. Smyth LCD, Rustenhoven J, Park TIH, et al. Unique and shared inflammatory profiles of human brain endothelia and pericytes. *J Neuroinflammation*. 2018;15(1):138. doi:10.1186/s12974-018-1167-8

21. Jain S, Rick JW, Joshi RS, et al. Single-cell RNA sequencing and spatial transcriptomics reveal cancer-associated fibroblasts in glioblastoma with protumoral effects. *J Clin Invest*. 2023;133(5):e147087. doi:10.1172/JCI147087

22. Andreatta M, Carmona SJ. UCell: Robust and scalable single-cell gene signature scoring. *Comput Struct Biotechnol J*. 2021;19:3796-3798. doi:10.1016/j.csbj.2021.06.043

23. Kueckelhaus J, Frerich S, Kada-Benotmane J, et al. Inferring histology-associated gene expression gradients in spatial transcriptomic studies. *Nat Commun*. 2024;15(1):7280. doi:10.1038/s41467-024-50904-x

24. Yu G, Wang LG, Han Y, He QY. clusterProfiler: an R Package for Comparing Biological Themes Among Gene Clusters. *OMICS: A Journal of Integrative Biology*. 2012;16(5):284-287. doi:10.1089/omi.2011.0118

Supporting Information: Figures

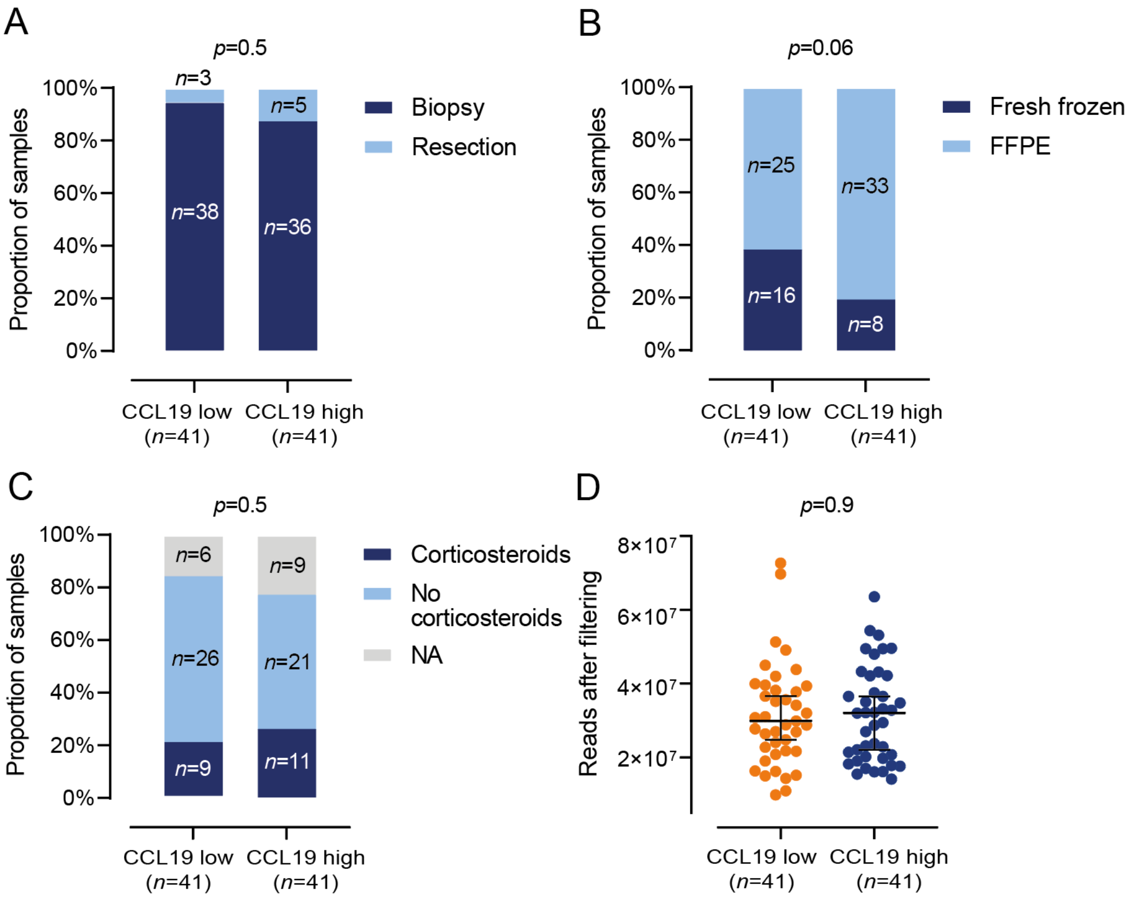

Supporting Information: Figure 1. Tissue characteristics of analyzed samples. (A) Bar graph comparing the proportion of samples obtained by biopsy or resection between the 'CCL19 high' and 'CCL19 low' subgroups. **(B)** Bar graph comparing the proportion of samples that were formalin-fixed paraffin-embedded (FFPE) or fresh frozen between the CCL19 subgroups. **(C)** Bar graphs comparing the proportion of tumor samples obtained with or without prior corticosteroid therapy between the CCL19 subgroups. NA; No data available. **(D)** Scatter plot showing the number of reads per sample after filtering for bulk RNA sequencing in the 'CCL19 high' and 'CCL19 low' subgroup'. Thick lines represent the median, errors bars show the 95% confidence intervals.

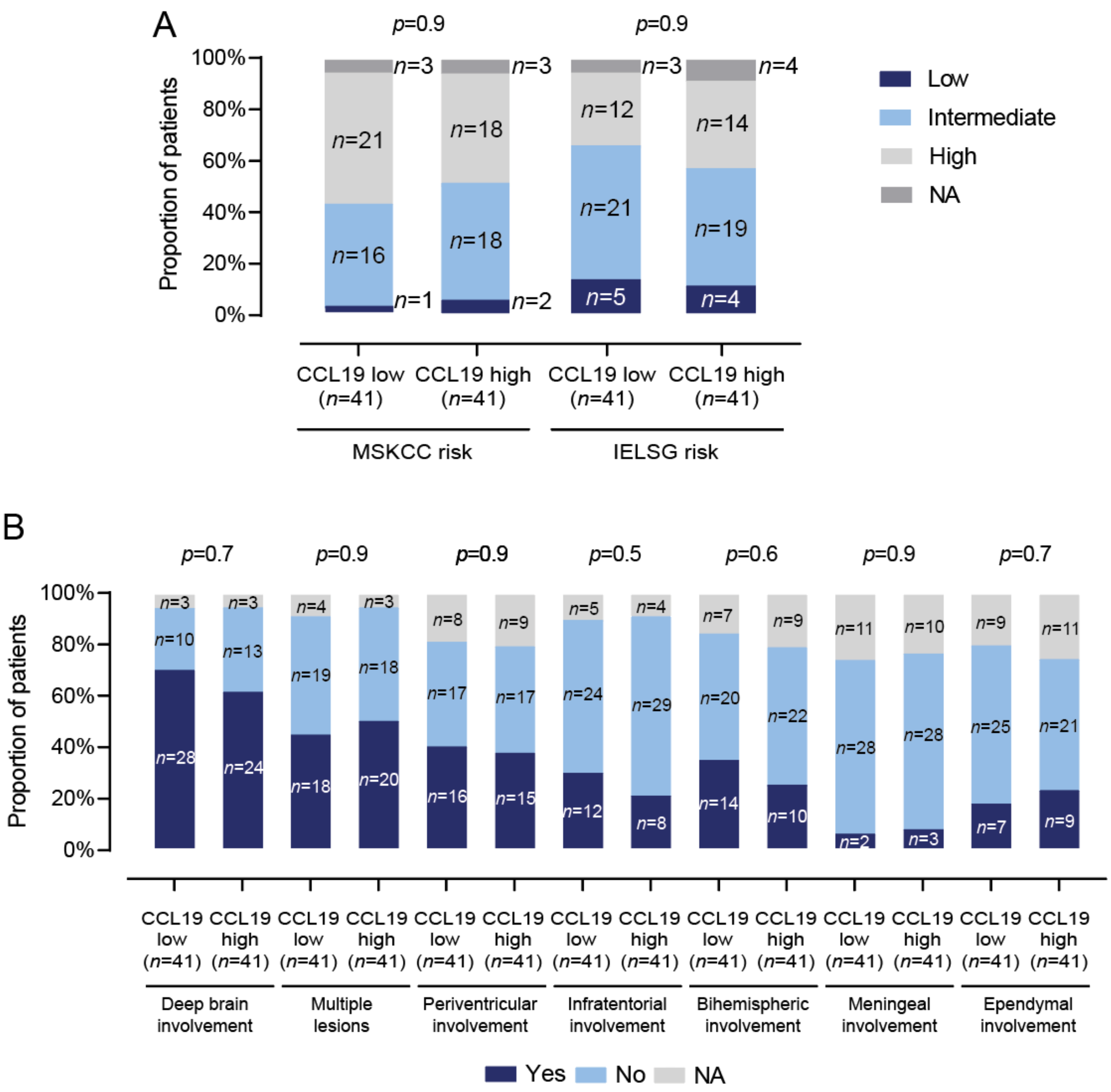

Supporting Information: Figure 2. Clinical and radiological characteristics of patients. (A) Bar graphs comparing the proportion of patients with low-, intermediate-, and high-risk status according to the MSKCC and IELSG scores between the CCL19 subgroups. NA, No data available. **(B)** Bar graphs comparing the occurrence of several radiological features between the 'CCL19 high' and 'CCL19 low' subgroup.

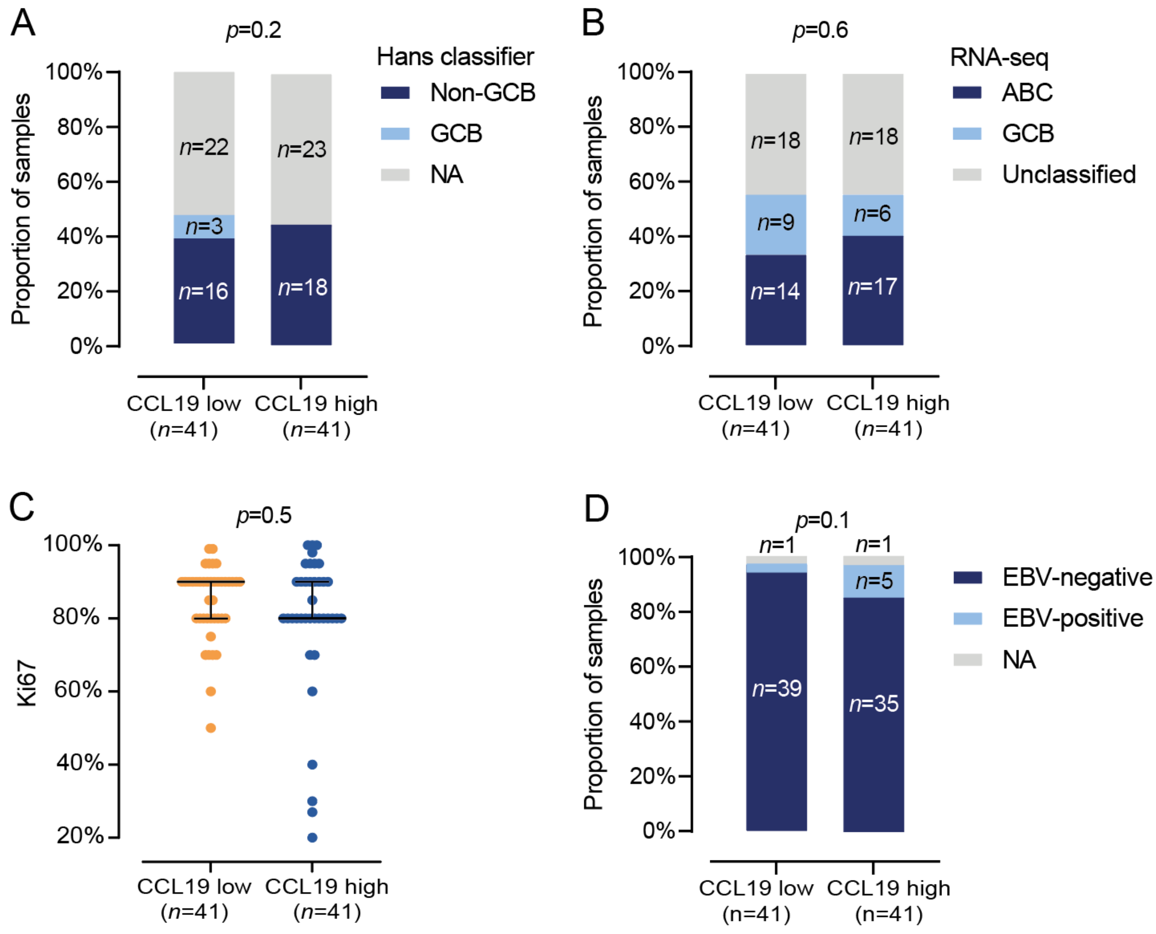

Supporting Information: Figure 3. Pathological characteristics of CNSL tumor specimens. (A) Bar graph comparing the proportion of germinal center B-cell (GCB) and non-GCB tumors between the CCL19 subgroups, defined according to the Hans classifier based on immunohistochemistry. NA, No data available. **(B)** Bar graph comparing the proportion of activated B-cell (ABC)-classified, GCB-classified, and unclassified CNSL by transcriptional profiling. **(C)** Scatter plot showing the percentage of Ki67-positive cells in ‘CCL19 high’ and ‘CCL19 low’ tumors. Thick lines represent the median, the error bars show the 95% confidence intervals. **(D)** Bar graph comparing the proportion of Epstein-Barr virus (EBV)-positivity and negativity between the ‘CCL19 high’ and ‘CCL19 low’ subgroup.

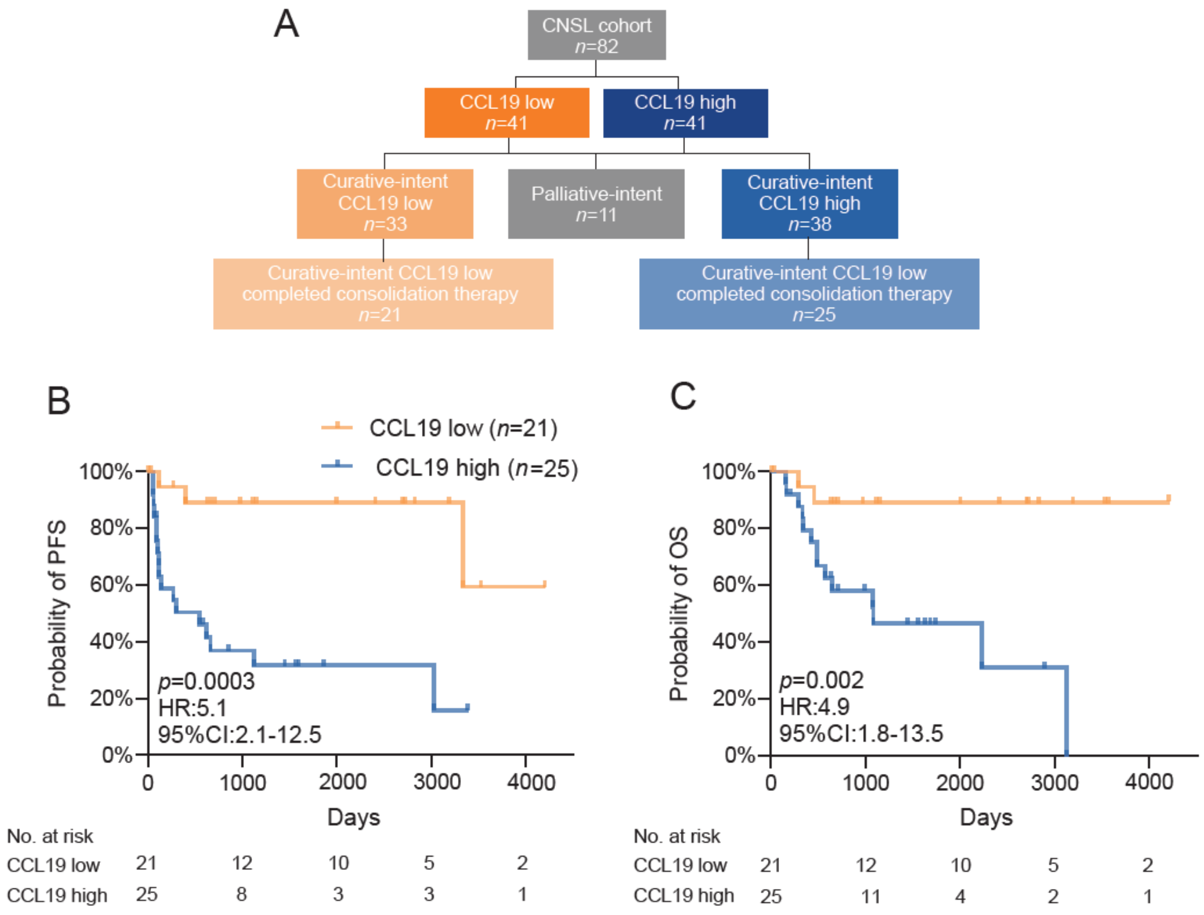

Supporting Information: Figure 4. Prognostic impact of CCL19 in patients who completed consolidation therapy. (A) Flowchart highlighting the patient cohorts. CNSL, Central nervous system lymphoma. Kaplan–Meier analyses of **(B)** PFS and **(C)** OS in patients of the 'CCL19 high' (light blue) and 'CCL19 low' group (light orange) who received curative-intent therapy and completed consolidation therapy. HR, hazard ratio; CI, confidence interval; No., number.

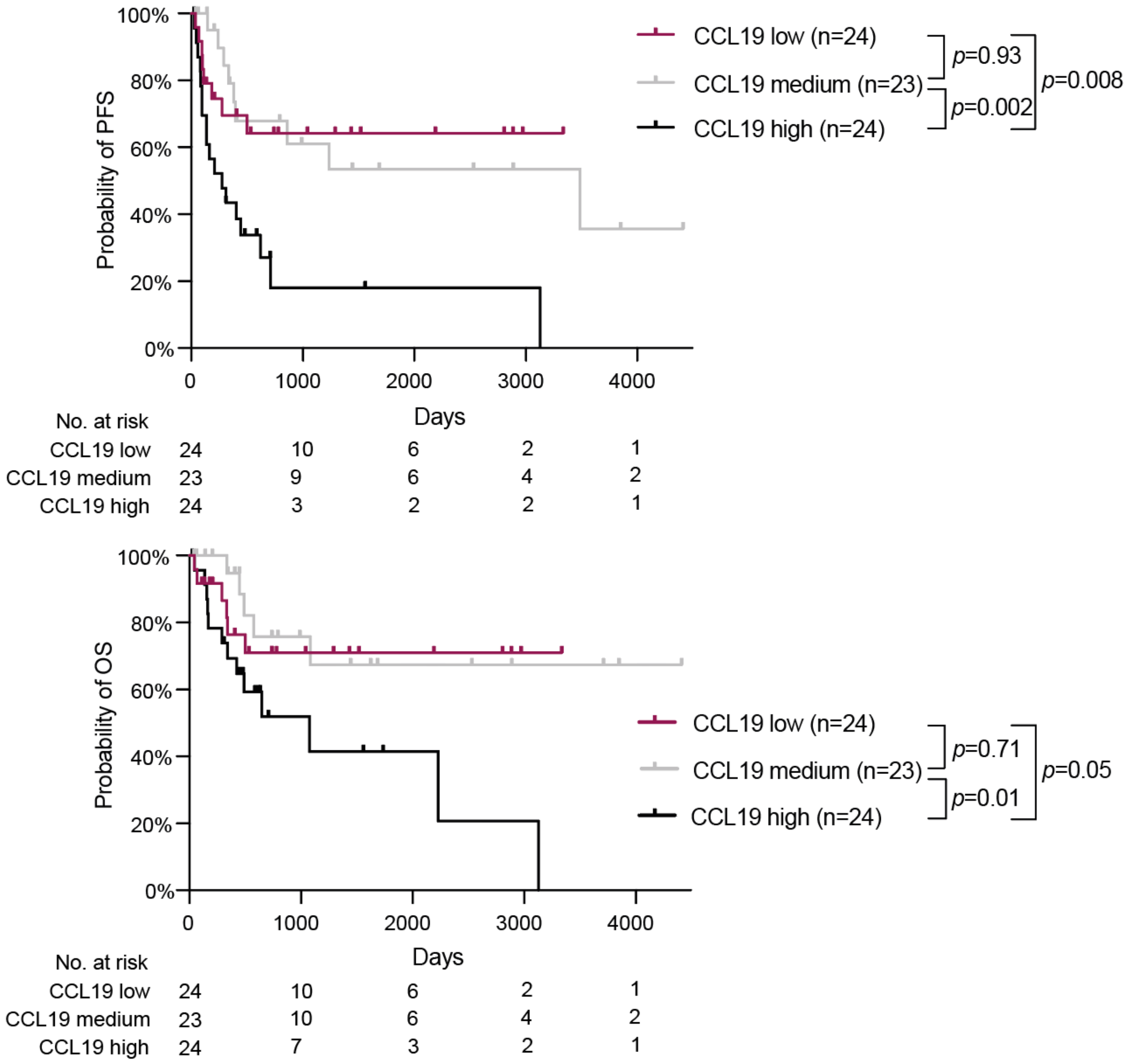

Supporting Information: Figure 5. CCL19 expression is associated with progression-free and overall survival. Kaplan–Meier survival analyses of progression-free survival (PFS, upper panel) and overall survival (OS, lower panel) in patients stratified into thirds according to CCL19 expression levels (low, medium, and high expression groups). No., number.

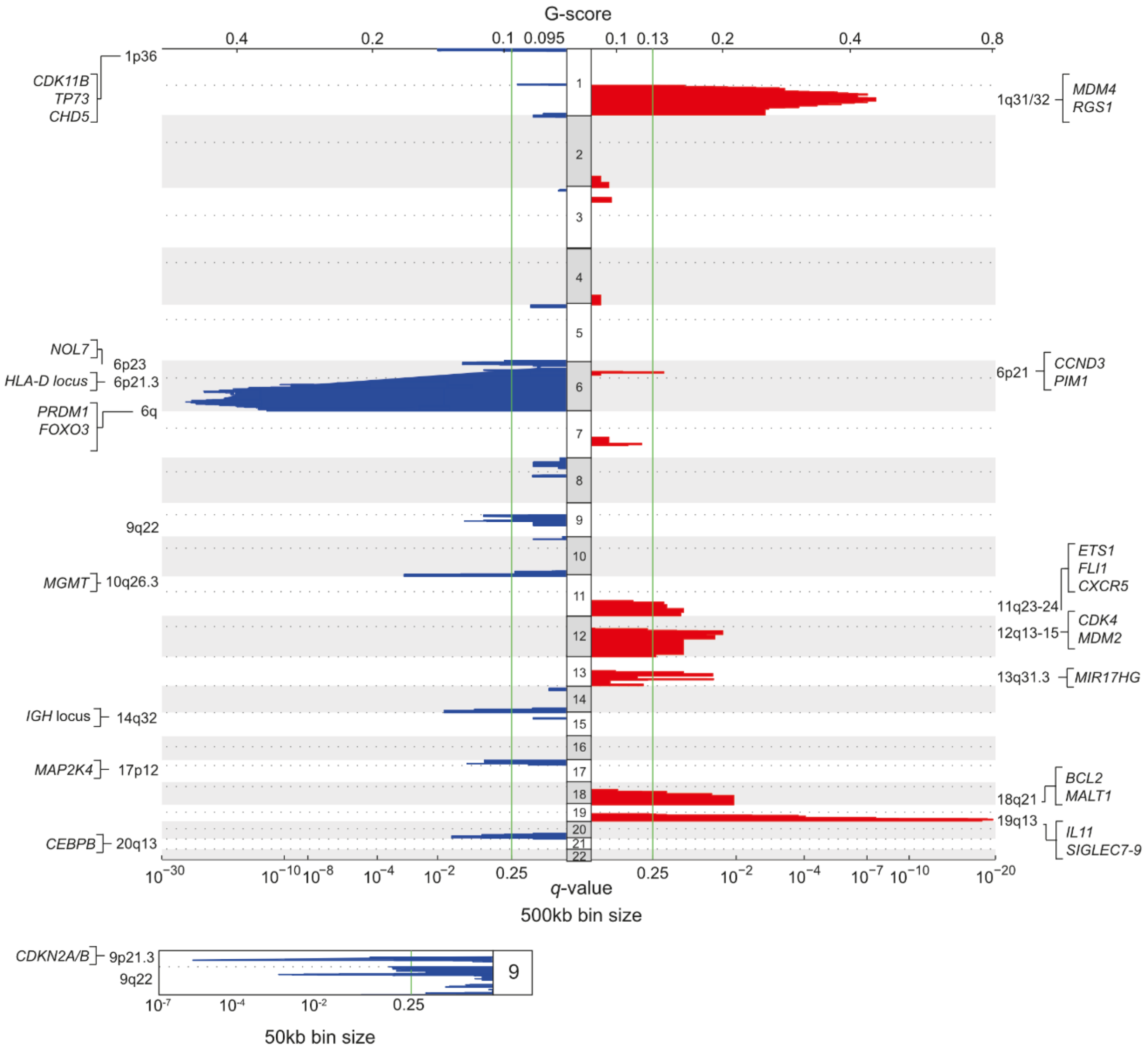

Supporting Information: Figure 6. CNA profile of the entire CNSL cohort. Copy number gains and copy number losses within the entire CNSL cohort, shown in GISTIC plots. Significantly altered regions/peaks are annotated using the respective cytoband. Selected candidate genes in these regions/peaks are stated. The analysis was performed using a 500 kb bin size, except for chromosome 9. Here, we applied a 50 kb bin size to identify small losses that involve *CDKN2A/B*. Significance was calculated using the GISTIC 2.0 permutation test with Benjamini-Hochberg correction for multiple testing. Green lines represent the significance thresholds.

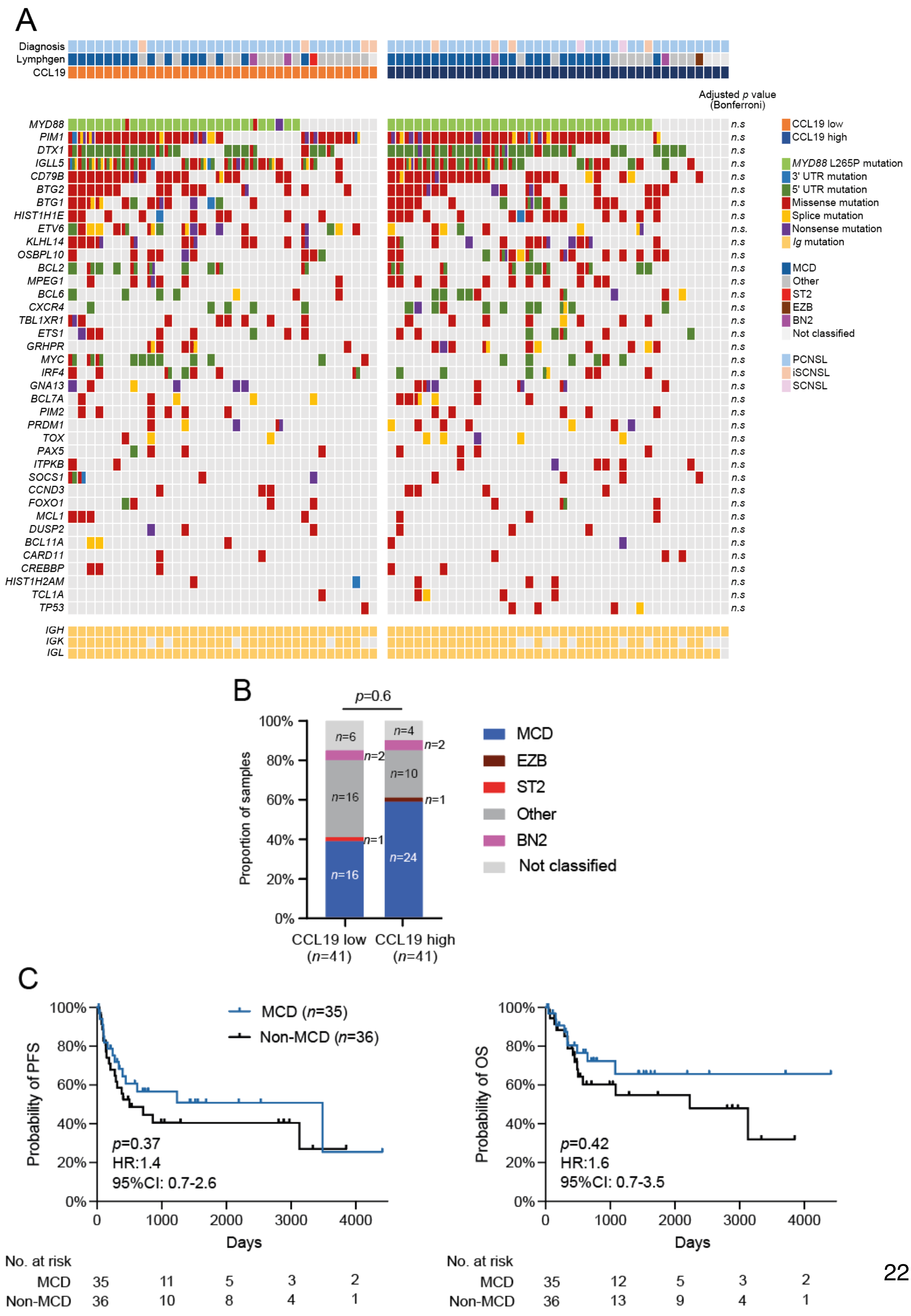
A
Diagnosis
Lymphgen
CCL19
Adjusted p value (Bonferroni)
MYD88
PIM1
DTX1
IGLL5
CD79B
BTG2
BTG1
HIST1H1E
ETV6
KLHL14
OSBPL10
BCL2
MPEG1
BCL6
CXCR4
TBL1XR1
ETS1
GRHPR
MYC
IRF4
GNA13
BCL7A
PIM2
PRDM1
TOX
PAX5
ITPKB
SOCS1
CCND3
FOXO1
MCL1
DUSP2
BCL11A
CARD11
CREBBP
HIST1H2AM
TCL1A
TP53
IGH
IGK
IGL
n.s
CCL19 low
CCL19 high
MYD88 L265P mutation
3' UTR mutation
5' UTR mutation
Missense mutation
Splice mutation
Nonsense mutation
Ig mutation
MCD
Other
ST2
EZB
BN2
Not classified
PCNSL
iSCNSL
SCNSL
B
p=0.6
100%
80%
60%
40%
20%
0%
Proportion of samples
n=6
n=2
n=16
n=1
n=16
n=4
n=2
n=10
n=1
n=24
CCL19 low (n=41)
CCL19 high (n=41)
MCD
EZB
ST2
Other
BN2
Not classified
C
MCD (n=35)
Non-MCD (n=36)
Probability of PFS
p=0.37
HR:1.4
95%CI: 0.7-2.6
0
1000
2000
3000
4000
Days
Probability of OS
p=0.42
HR:1.6
95%CI: 0.7-3.5
No. at risk
MCD 35 11 5 3 2
Non-MCD 36 10 8 4 1
No. at risk
MCD 35 12 5 3 2
Non-MCD 36 13 9 4 1

Supporting Information: Figure 7. Somatic mutation profiles and LymphGen classification of the entire cohort. (A) Case-level mutational profiles of the entire CNSL cohort, profiled by CAPP-Seq. Each column represents a tumor sample, each row represents a gene. Genes with at least five recurrent mutations in the cohort are shown. Genotyping grids were generated for the 'CCL19 high' (left) and 'CCL19 low' group (right) separately and were manually clustered to highlight mutation co-occurrence. The diagnosis, LymphGen subtypes, and CCL19 subgroups are color-coded in the first three rows. The Benjamin-Hochberg adjusted *p*-values comparing the frequency of mutations in the 'CCL19 high' and 'CCL19 low' subgroup are provided for each gene. UTR, untranslated region; Ig, immune globulin; PCNSL; primary central nervous system lymphoma; iSCNSL, isolated secondary central nervous system lymphoma; SCNSL, secondary central nervous system lymphoma; *n.s.*, not significant. **(B)** Bar graph comparing the proportion of LymphGen subtypes between the 'CCL19 high' and 'CCL19 low' subgroups. **(C)** Kaplan–Meier analyses of PFS and OS in patients with tumors classified as MCD (blue) compared to patients with tumor classified as Non-MCD (black). HR, hazard ratio; CI, confidence interval; No., number.

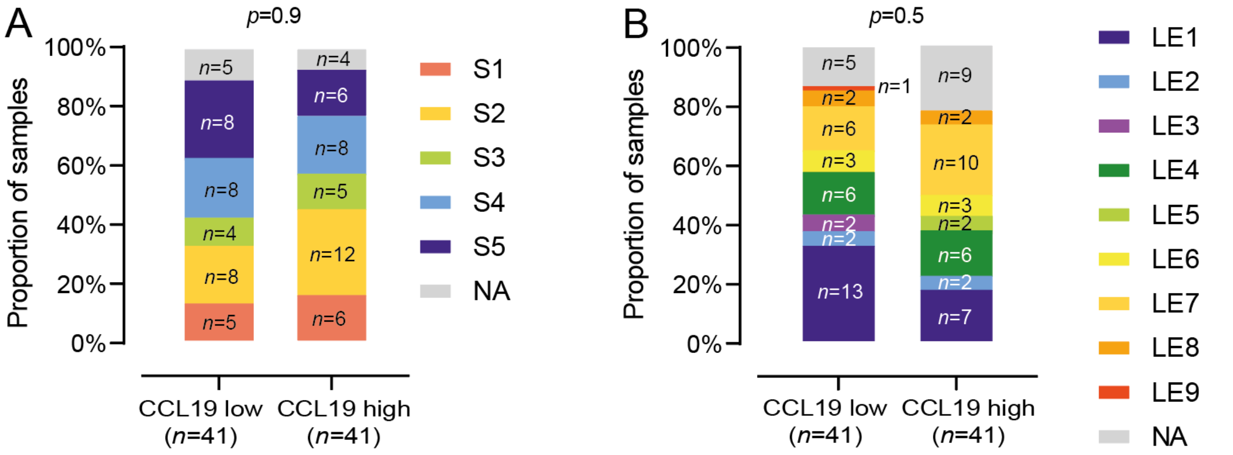

Supporting Information: Figure 8. B-cell states and lymphoma ecotypes. (A) Bar graph comparing the proportion of B-cells states (S1-S5) between the 'CCL19 high' and the 'CCL19 low' subgroup. NA, not assigned. **(B)** Bar graph comparing the proportion of lymphoma ecotypes (LE1-LE9) between the CCL19 subgroups.

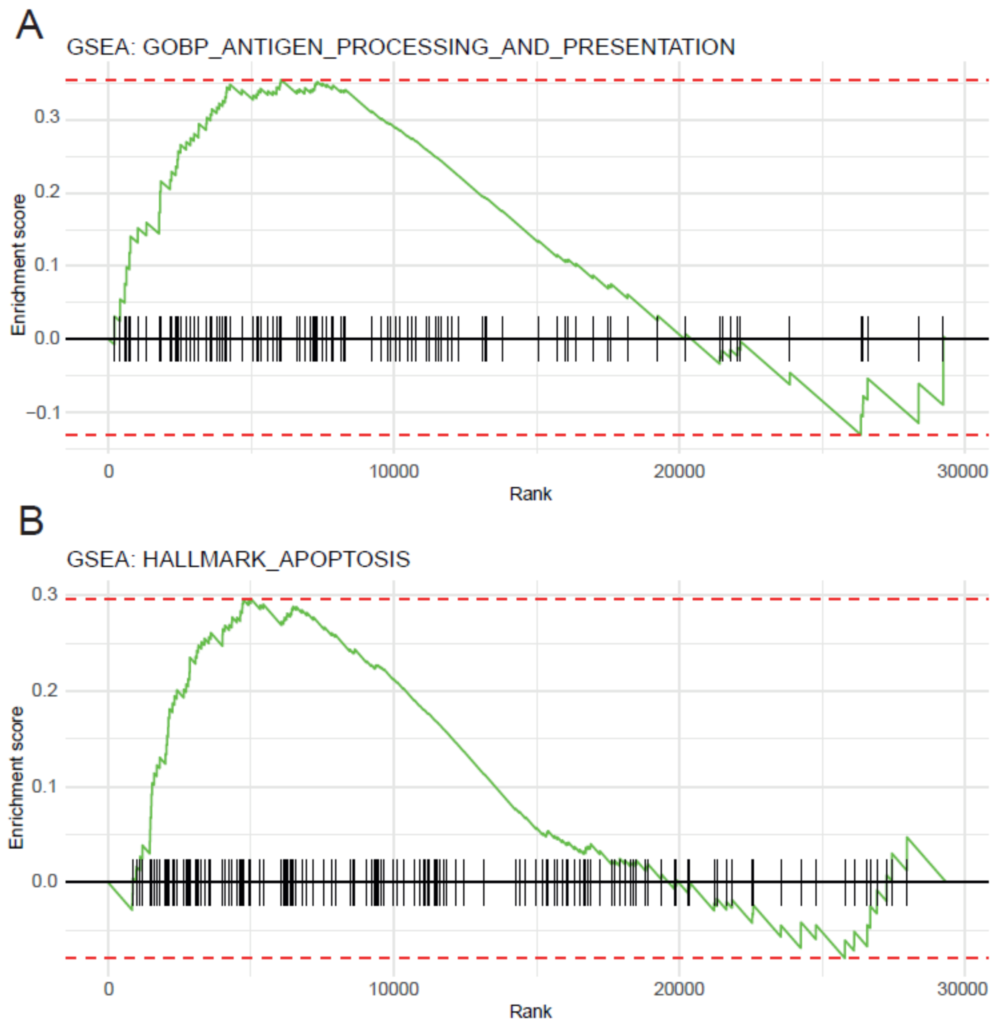

Supporting Information: Figure 9. Gene set enrichment analysis of the 'CCL19 low' subgroup. Enrichment plot for (**A**) GO Biological Process gene set "ANTIGEN_PROCESSING_AND_PRESENTATION" and (**B**) HALLMARK gene set "APOPTOSIS", both revealing an enrichment of expression in the 'CCL19 low' subgroup The normalized enrichment scores (NES) for **(A)** was 1.5 (p=0.01) and for **(B)** 1.3 (p=0.04). The green lines represent the running enrichment score (ES) across the ranked gene lists, with vertical black lines indicating the positions of genes from the respective gene sets.

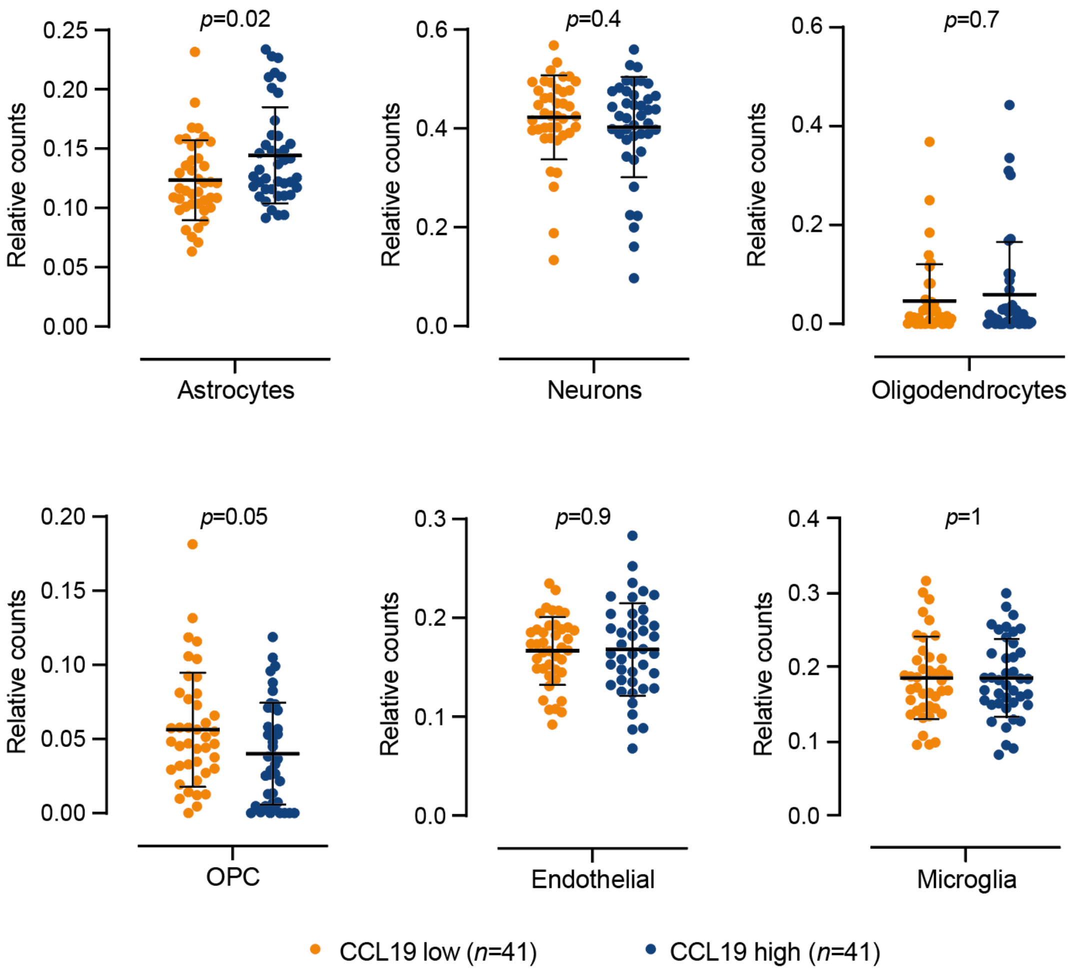

Supporting Information: Figure 10. CIBERSORTx deconvolution results for central nervous system specific cell populations. Scatter plots showing the relative count of each cell type in each CCL19 subgroup. Thick lines represent the median and error bars show the 95% confidence intervals.

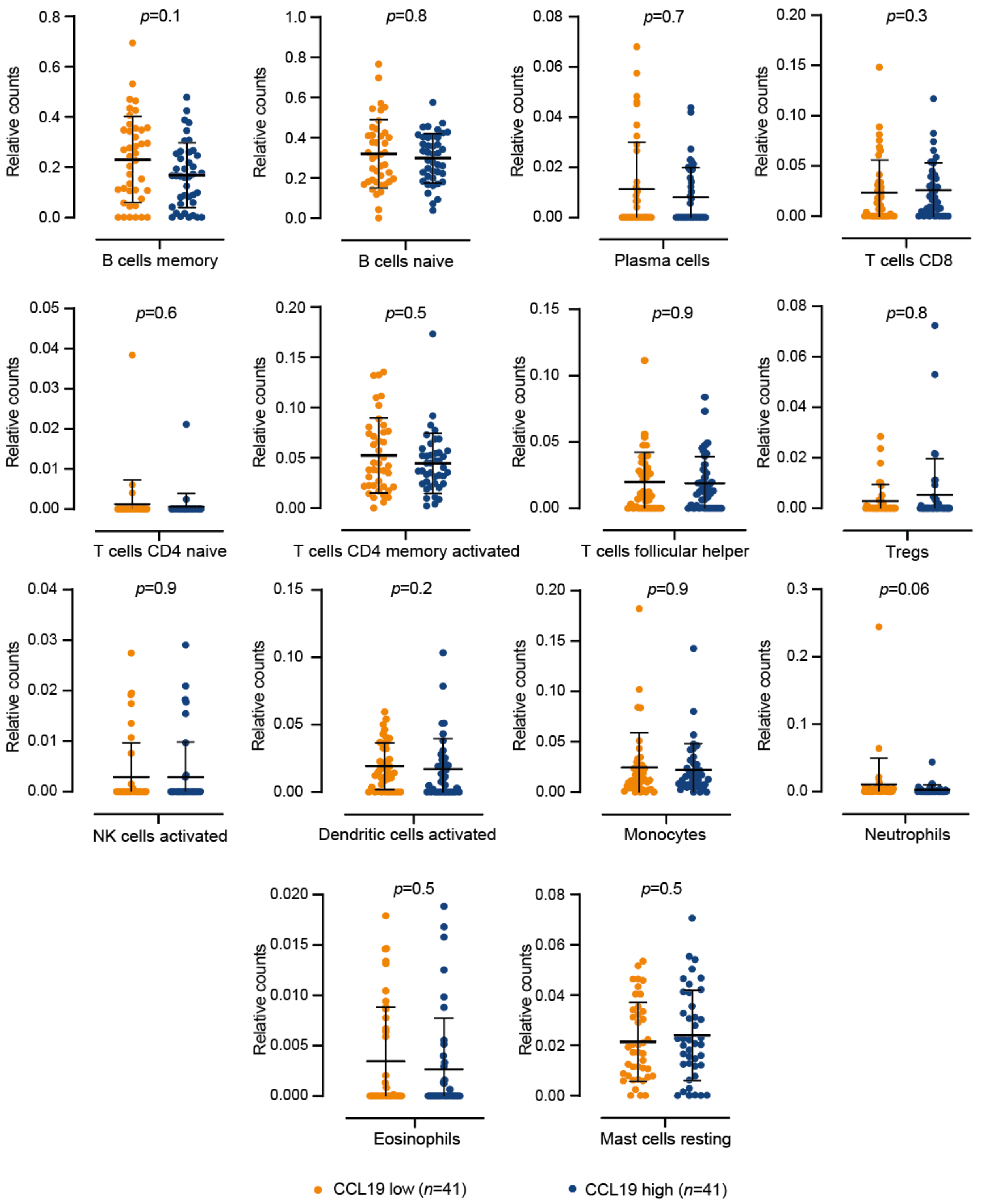
p=0.1
B cells memory
p=0.8
B cells naive
p=0.7
Plasma cells
p=0.3
T cells CD8
p=0.6
T cells CD4 naive
p=0.5
T cells CD4 memory activated
p=0.9
T cells follicular helper
p=0.8
Tregs
p=0.9
NK cells activated
p=0.2
Dendritic cells activated
p=0.9
Monocytes
p=0.06
Neutrophils
p=0.5
Eosinophils
p=0.5
Mast cells resting
Relative counts
CCL19 low (n=41)
CCL19 high (n=41)

Supporting Information: Figure 11. CIBERSORTx deconvolution results for immune cell types. Scatter plots showing the relative count of each cell type in each CCL19 subgroup. Cell types that were not detected in all samples are not depicted. Cell types with significantly different counts are shown in Figure 2. Thick line, median; Error bars, 95% confidence interval; Dots, single values.

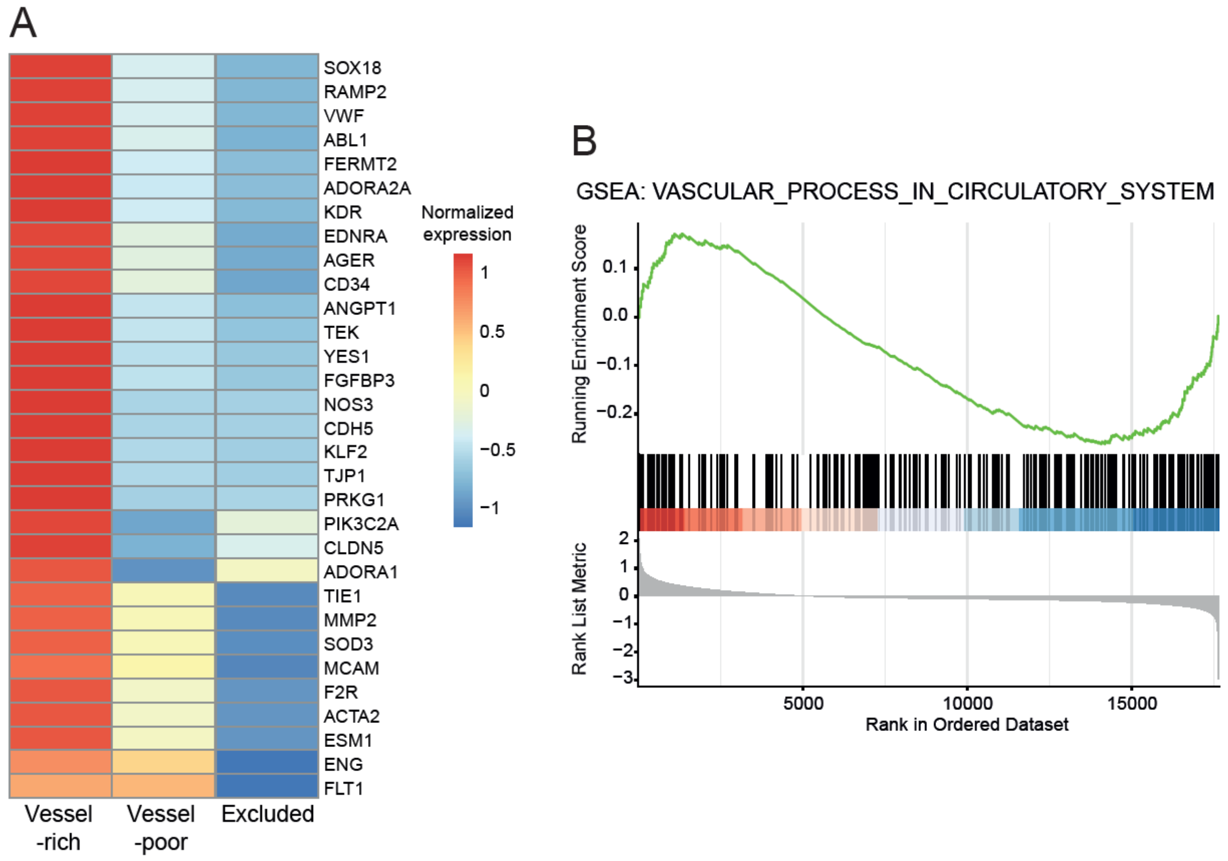

Supporting Information: Figure 12. Enrichment of vessel-associated gene signatures in 'vessel-rich' spots. (**A**) Heatmap of the mean normalized expression of vessel-associated genes in 'vessel-rich', 'vessel-poor', and 'excluded' spots of the spatial transcriptomics data sets. (**B**) Gene Set Enrichment Analysis (GSEA) for the GO Biological Process gene set "VASCULAR_PROCESS_IN_CIRCULATORY_SYSTEM", showing depletion of this gene set in the vessel-poor subgroup (Normalized enrichment score (NES)=-1.2, p=0.04). The green line represents the running enrichment score (ES); black vertical lines indicate the positions of genes from the gene set in the ranked list. The color bar below shows the rank metric, with red indicating genes highly expressed in the vessel-rich group and blue indicating genes enriched in the vessel-poor group.

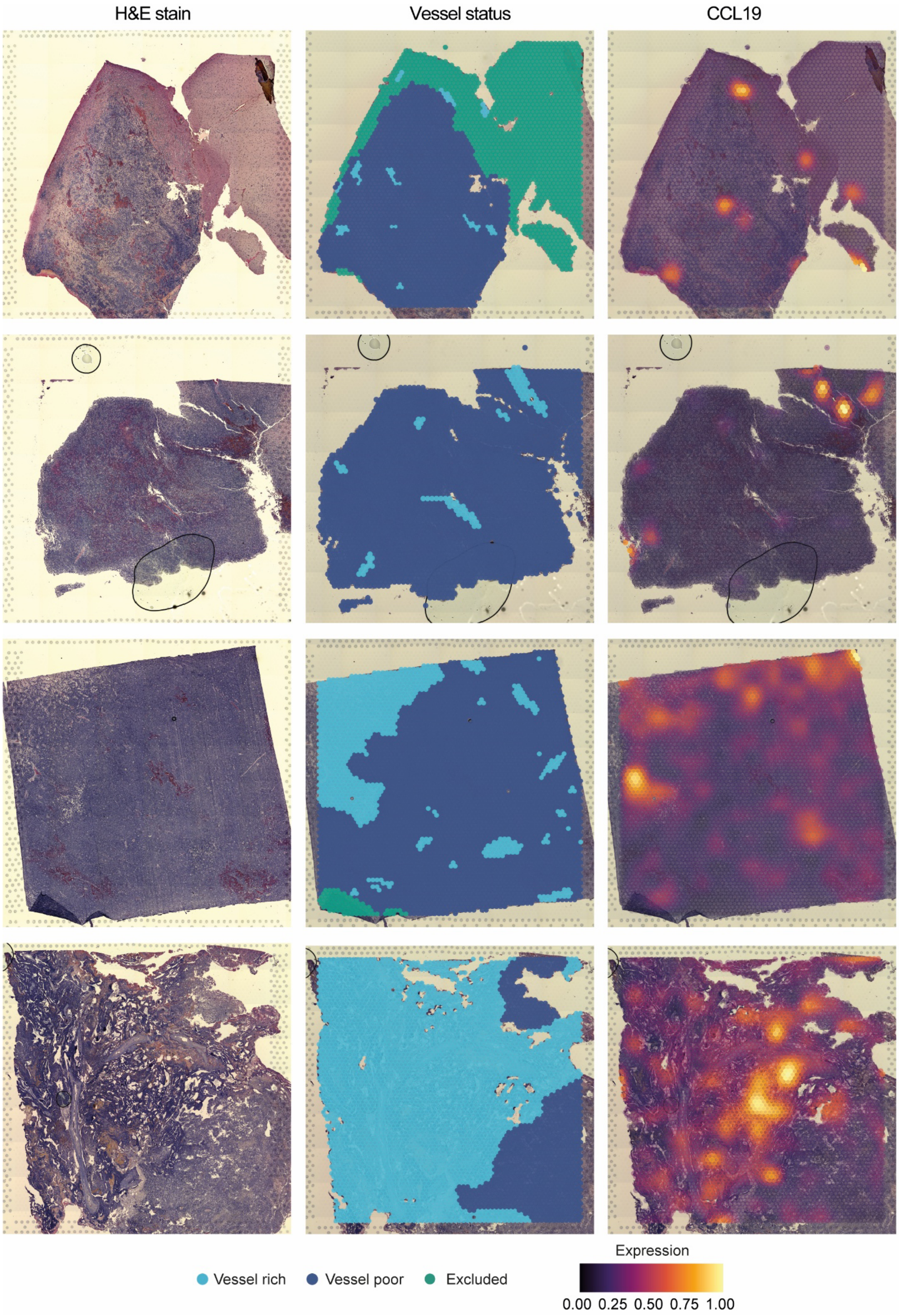
H&E stain
Vessel status
CCL19
Vessel rich
Vessel poor
Excluded
Expression
0.00
0.25
0.50
0.75
1.00

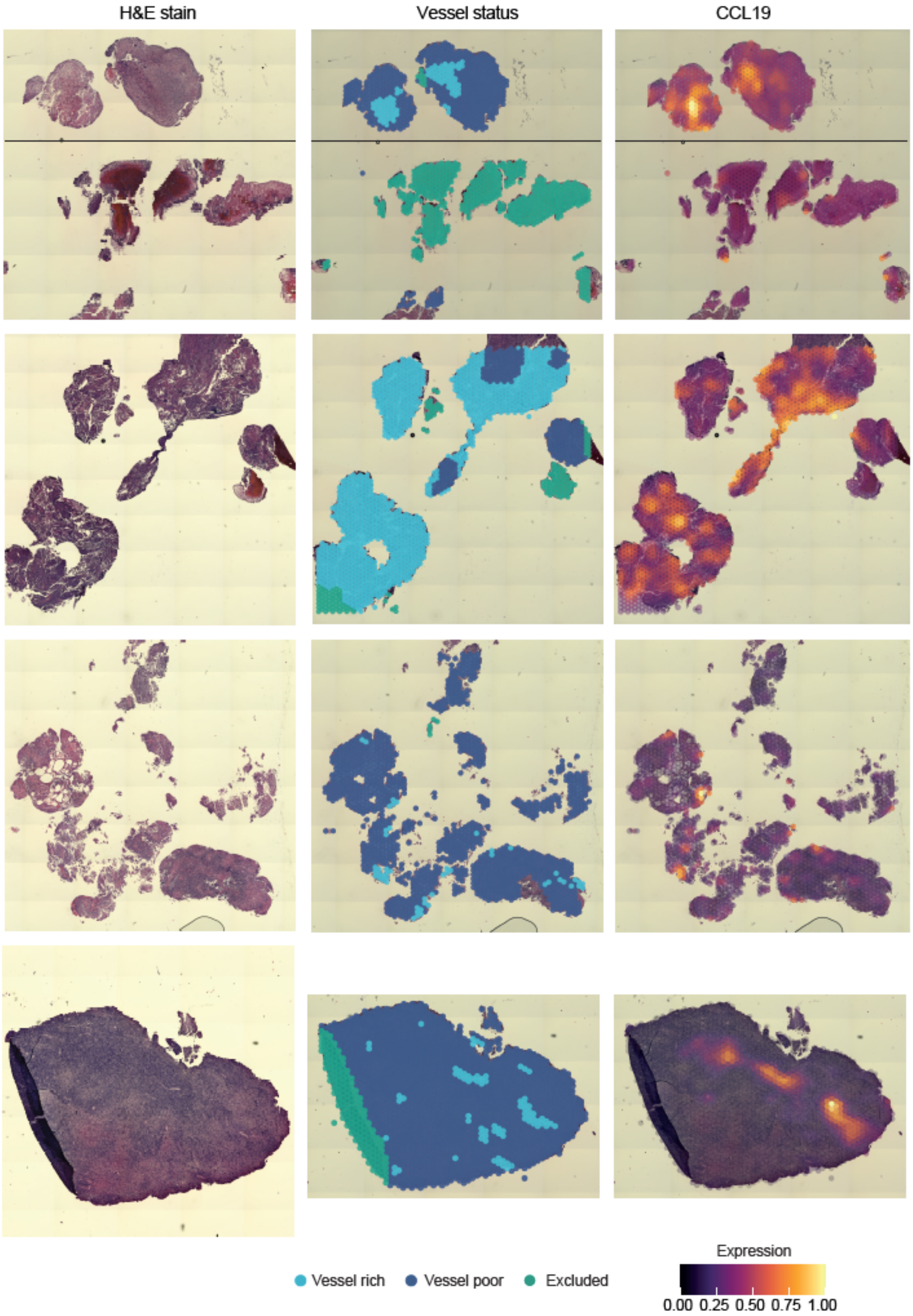
H&E stain
Vessel status
CCL19
Vessel rich
Vessel poor
Excluded
Expression
0.00 0.25 0.50 0.75 1.00

Supporting Information: Figure 13. Case-level Hematoxylin and Eosin (H&E)-stained images, image segmentation, and CCL19 expression of tumor tissue sections. Original H&E-stained images of all tissue sections used for 10x Visium spatial transcriptomics assays are shown on the left. Segmentation of spots into ‘vessel-rich’ and ‘vessel-poor’ are shown in the middle. The projection of CCL19 expression is shown on the right. The horizontal black line separates slices from two distinct tumor samples.

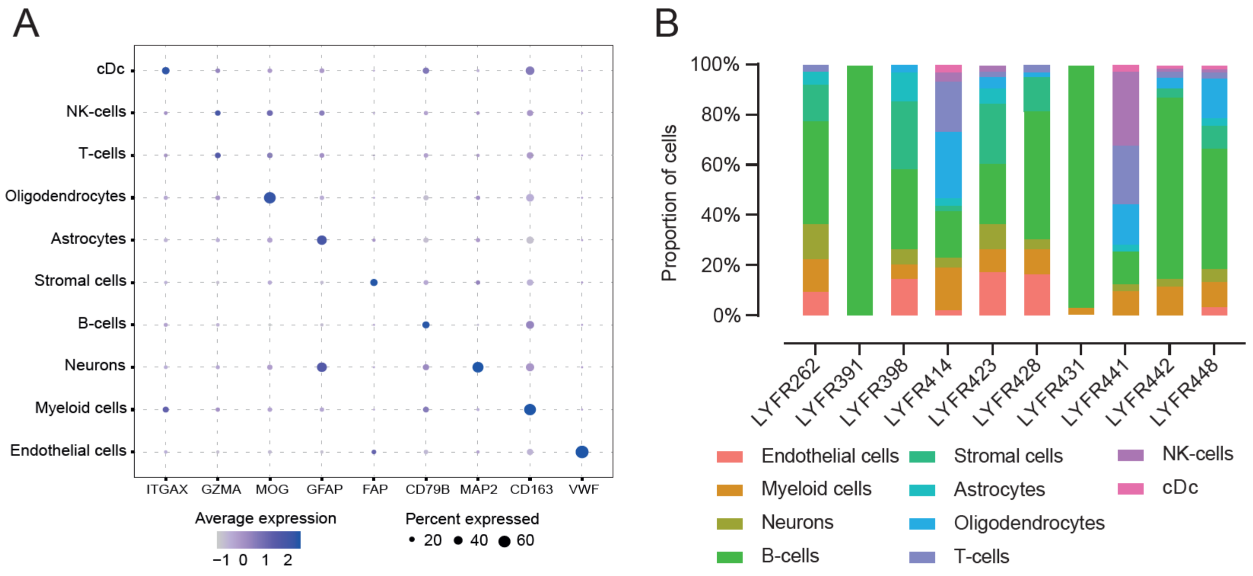

Supporting Information: Figure 14. Composition of the CNSL single-nucleus RNA sequencing dataset. (A) Dot plot of marker gene expression levels across annotated clusters shown in Figure 4A. Each column represents a cell type-specific marker gene, each row represents an annotated cluster/cell type. cDC, conventional dendritic cells. **(B)** Bar graph showing the proportion of each cell type within the ten tumor samples.

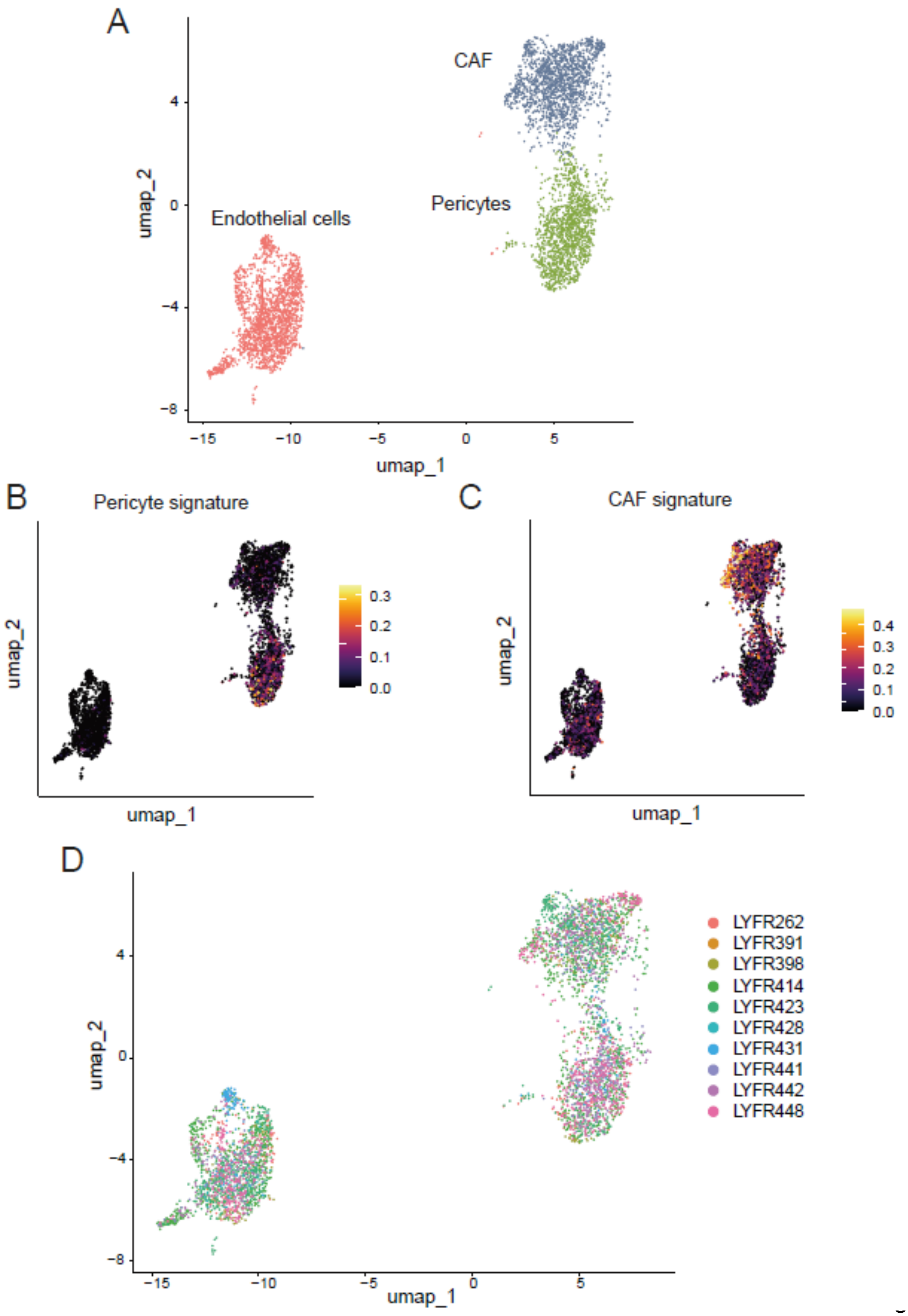
A
CAF
Pericytes
Endothelial cells
umap_2
umap_1
B
Pericyte signature
C
CAF signature
D
LYFR262
LYFR391
LYFR398
LYFR414
LYFR423
LYFR428
LYFR431
LYFR441
LYFR442
LYFR448

Supporting Information: Figure 15. The vascular compartment of CNSL specimens. **(A)** Uniform Manifold Approximation and Projection (UMAP) plot showing all cell types of the vascular compartment. CAF, cancer-associated fibroblasts. **(B,C)** UMAP plots highlighting the pericyte signature and CAF signature within the clusters of the vascular compartment. **(D)** UMAP plot displaying the same clusters, color-coded by tumor sample.

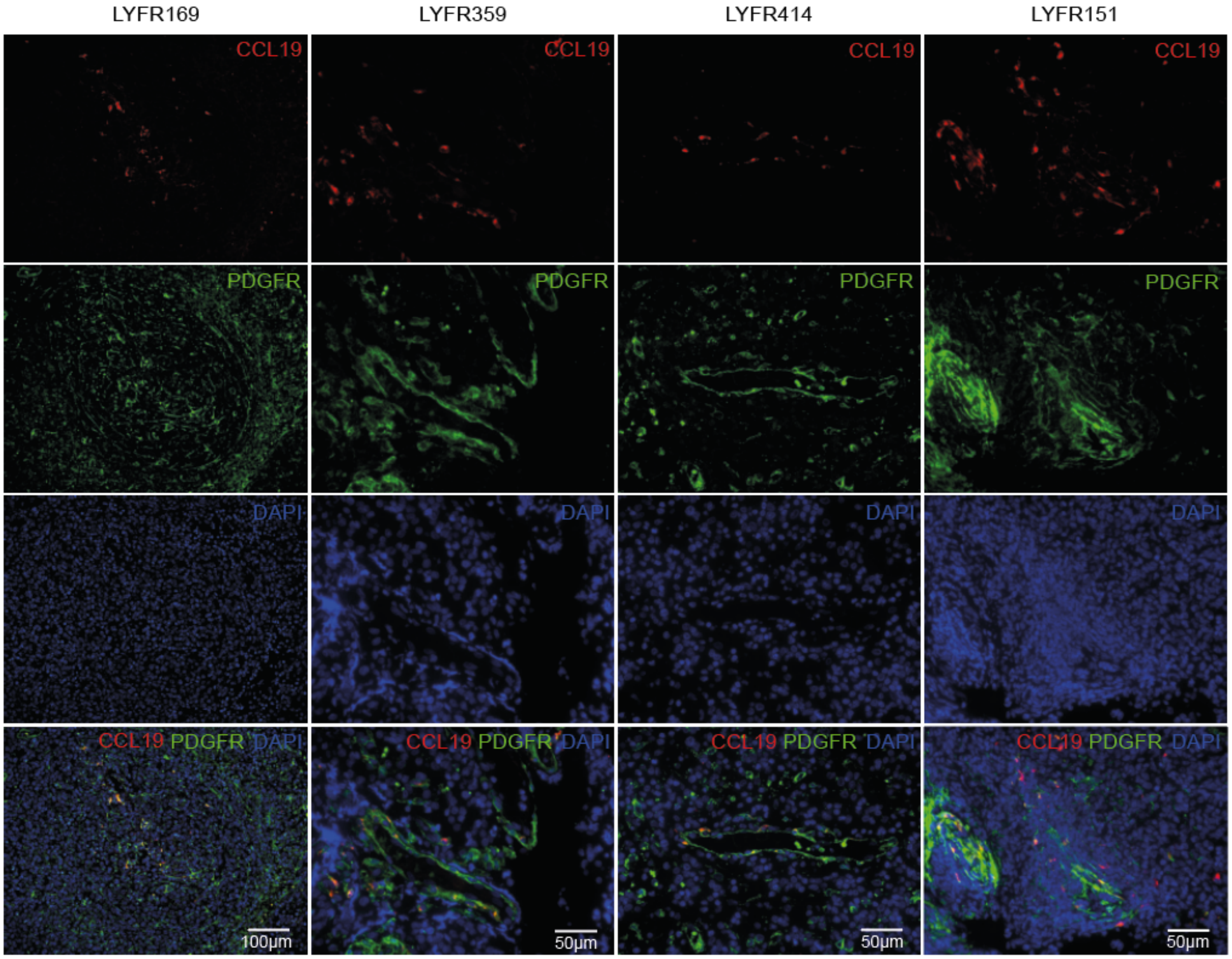

Supporting Information: Figure 16. Immunohistochemical co-localization of CCL19 and pericyte marker PDGFR. Immunohistochemistry of four CNSL tumor specimens, showing CCL19 in red (top row), the pericyte marker PDGFR in green (second row), and individual nuclei in blue (DAPI, third row). The composite image CCL19 and PDGFR is shown in the fourth row. Importantly, co-expression of CLL19 and PDGFR is indicated by the color yellow. Each tumor case is represented by one column. PDGFR, Platelet-Derived Growth Factor Receptor; DAPI, 4′,6-Diamidino-2-phenylindol.

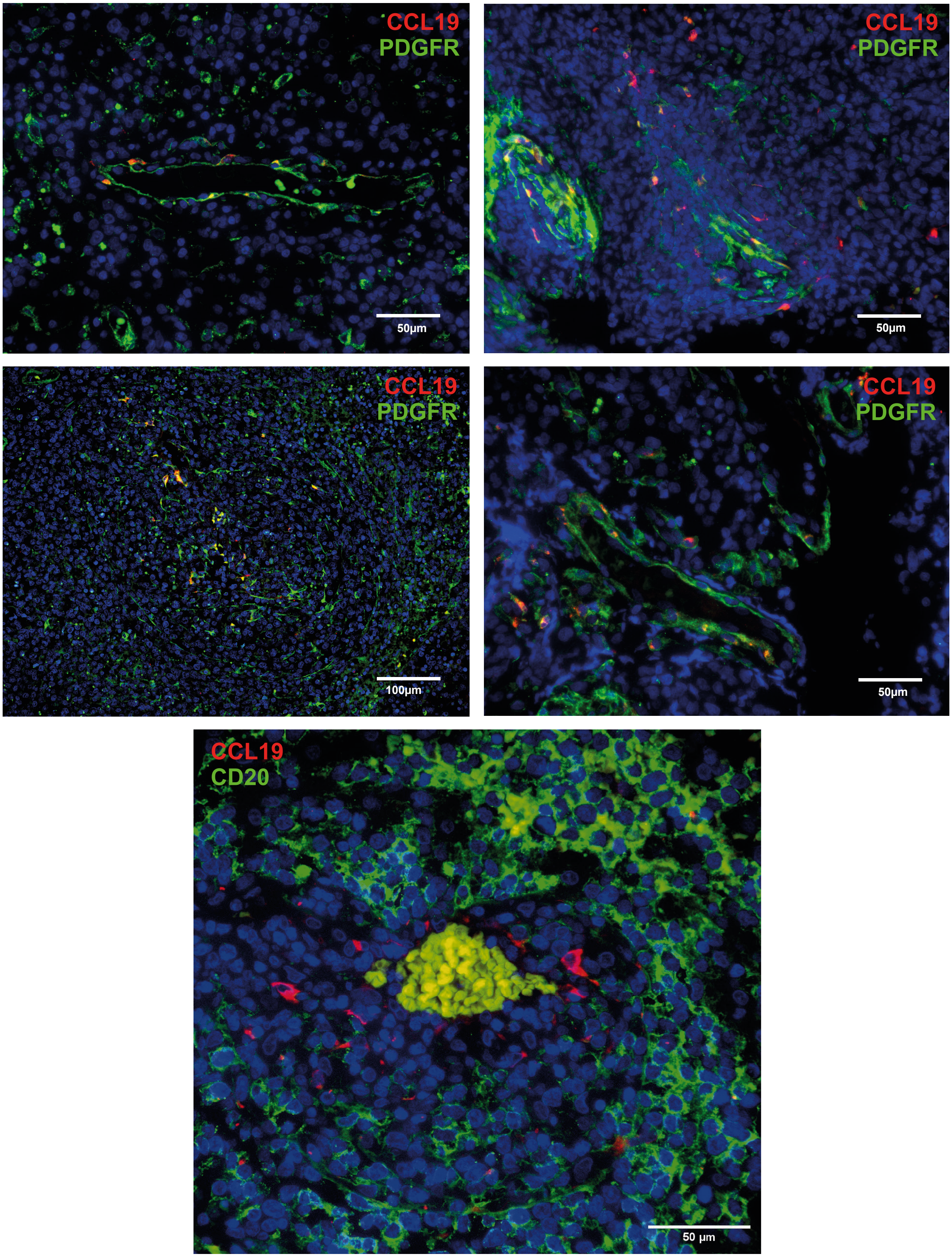
CCL19
PDGFR
50µm
CCL19
PDGFR
50µm
CCL19
PDGFR
100µm
CCL19
PDGFR
50µm
CCL19
CD20
50 µm

Supporting Information: Figure 17. Immunohistochemical co-localization of CCL19 and pericyte marker PDGFR (higher resolution) as well as B cell marker CD20.

Same representative immunohistochemistry images as shown in Supporting Information: Figure 15 but higher resolution of the four CNSL tumor specimens demonstrating co-localization of CCL19 in red and PDGFR in green (top panels). The bottom image displays the co-localization of CCL19 in red and CD20 in green (bottom). Of note, the erythrocytes in the middle of the image represent an artefact with strong auto-fluorescence that was omitted in the analysis. DAPI (′,6-Diamidino-2-phenylindol) nuclear stain in blue. PDGFR, Platelet-Derived Growth Factor Receptor.

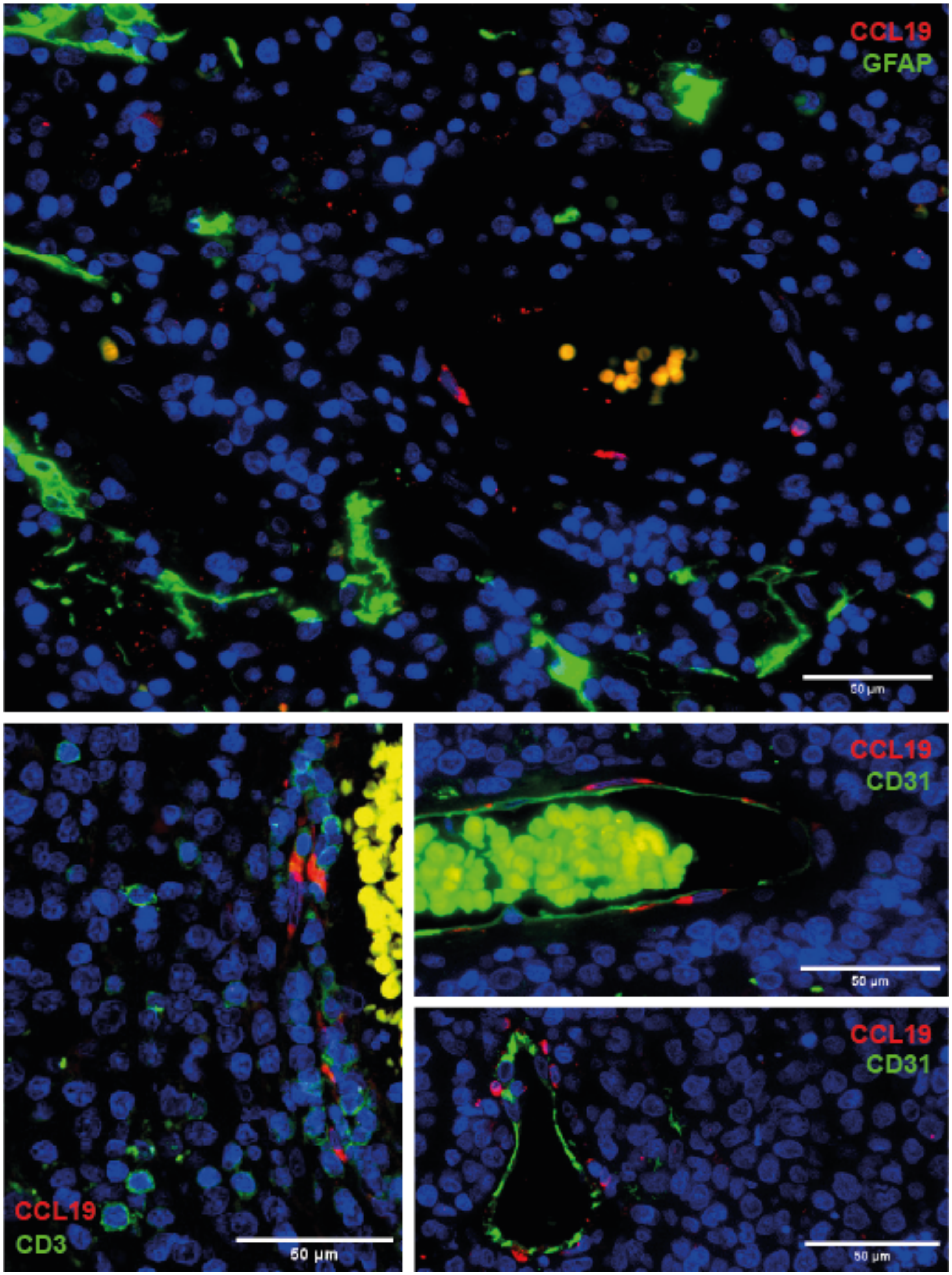
CCL19
GFAP
50 µm
CCL19
CD3
50 µm
CCL19
CD31
50 µm
CCL19
CD31
50 µm

Supporting Information: Figure 18. Immunohistochemical co-localization of CCL19 and the astrocyte marker GFAP, T cell marker CD3, and endothelial cell marker CD31.

Representative immunohistochemistry images showing the co-localization of CCL19 in red and GFAP (green; top), CD3 (green, bottom right) and CD31(green, bottom left). DAPI (′,6-Diamidino-2-phenylindol) nuclear stain in blue. GFAP, glial fibrillary acidic protein.

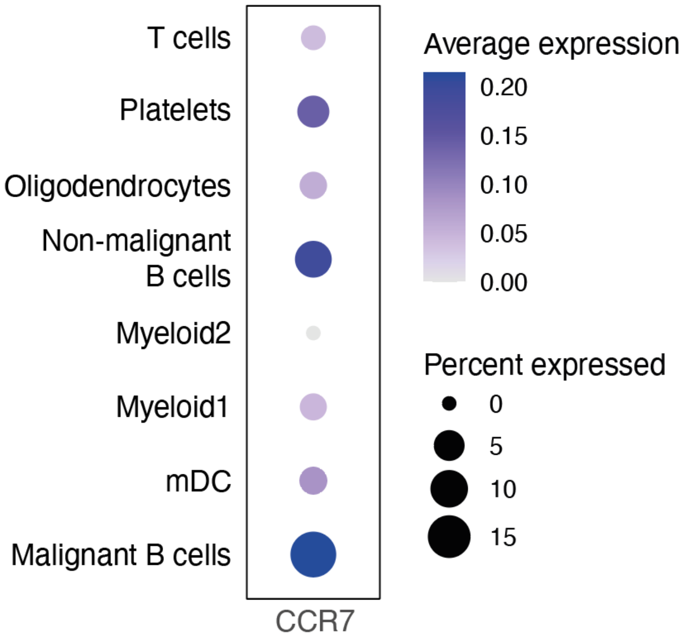

Supporting Information: Figure 19. CCR7 expression in an independent cohort of PCNSL samples (Heming et al., 2022)

Dot plot showing CCR7 expression within major cell types identified by single-cell RNA sequencing in an independent cohort of PCNSL cases.

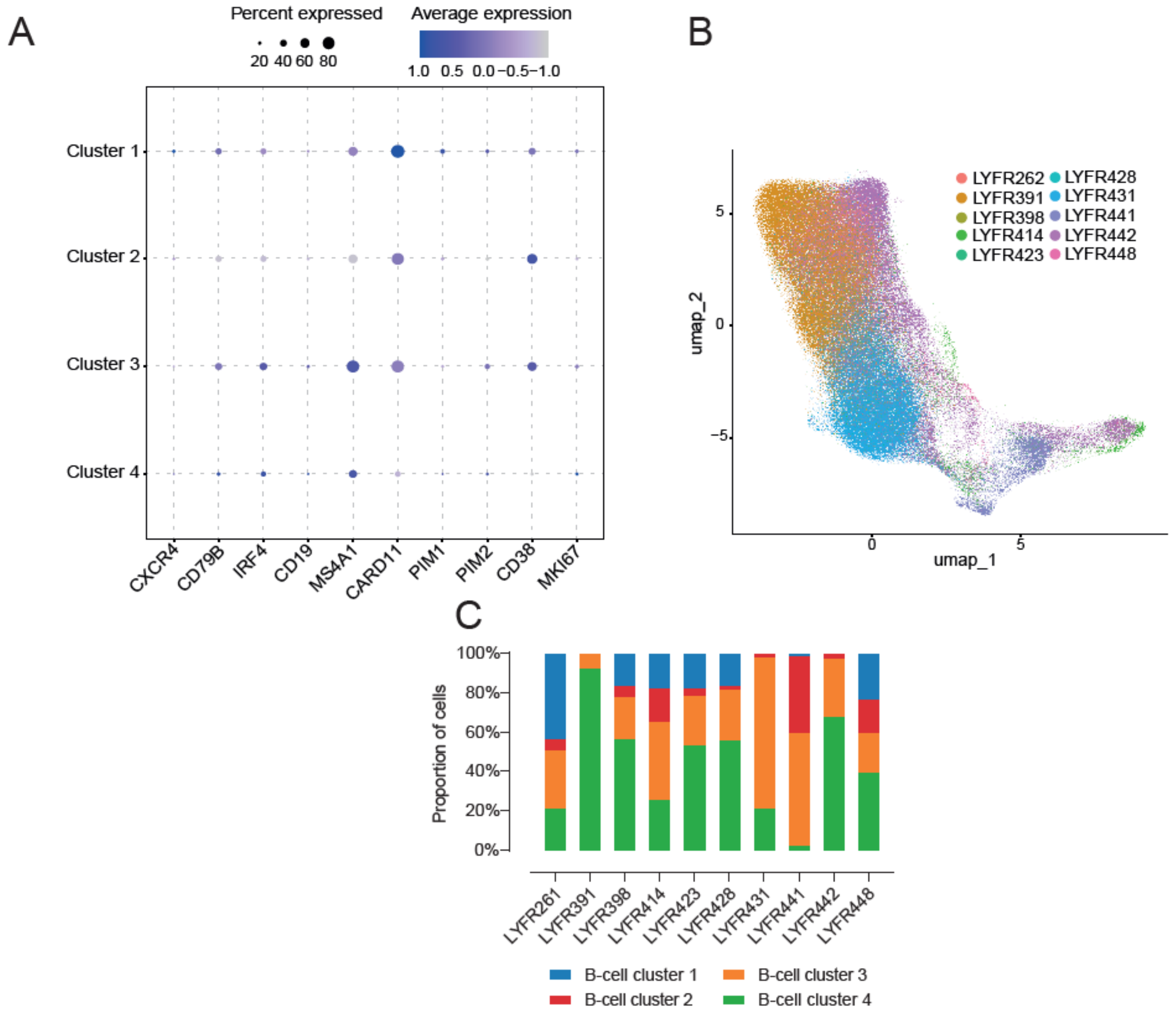

Supporting Information: Figure 20. The B-cell compartment of CNSL specimens. (A) Dot plot of B-cell marker gene expression levels across annotated clusters as shown in Figure 5E. **(B)** Uniform Manifold Approximation and Projection (UMAP) plot of the B-cell subclusters, colored by tumor sample. **(C)** Bar graph showing the proportion of each B-cell cluster in each tumor sample.

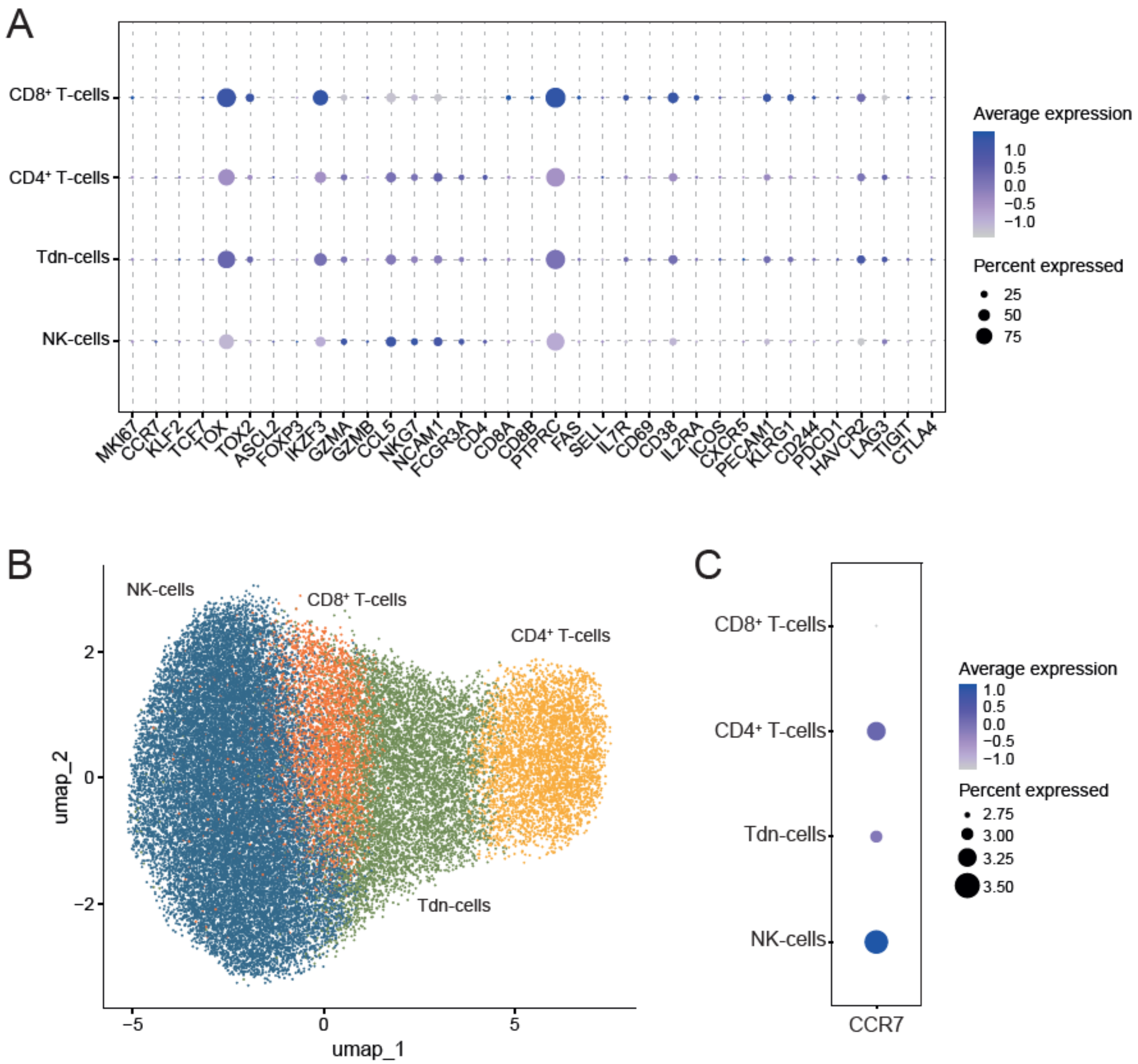

Supporting Information: Figure 21. The T-/NK-cell compartment of CNSL specimens. (A) Dot plot of T/NK-cell marker gene expression levels across annotated T/NK-cell clusters. Tdn, T cells double negative. **(B)** Uniform Manifold Approximation and Projection (UMAP) plot of the T/NK-cell compartment. The clusters are color-coded and annotated. **(C)** Dot plot showing the CCR7 expression levels in the T/NK-cell clusters.

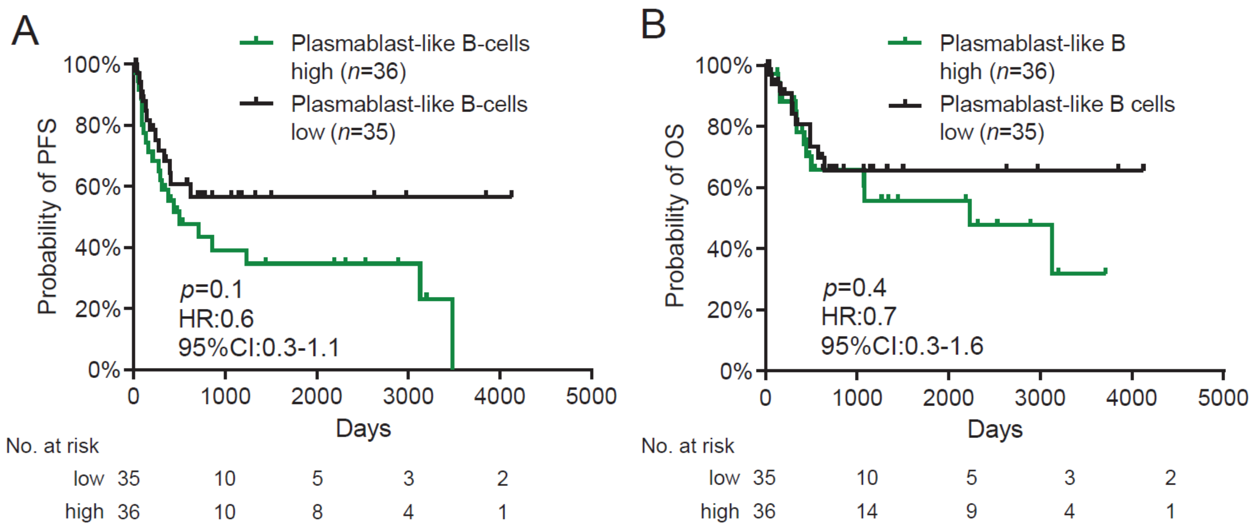

Supporting Information: Figure 22. Prognostic relevance of plasmablast-like B cells. Kaplan–Meier analyses of **(A)** PFS and **(B)** OS in patients with a plasmablast-like B-cells count above the median (green) compared to patients with a plasmablast-like B-cell count below the median (black). HR, hazard ratio; CI, confidence interval; No., number.
